# Context-aware multimodal AI navigates hidden pathways in five centuries of art evolution

**DOI:** 10.1073/pnas.2517969123

**Published:** 2026-07-24

**Authors:** Jin Kim, Byunghwee Lee, Taekho You, Jinhyuk Yun

**Affiliations:** ^a^ Department of Intelligent Semiconductors, Soongsil University, Seoul 06978, Republic of Korea; ^b^ School of Digital Humanities & Computational Social Sciences, Korea Advanced Institute of Science and Technology, Daejeon 34141, Republic of Korea; ^c^ School of Data Science, University of Virginia, Charlottesville, VA 22903; ^d^Luddy School of Informatics, Computing, and Engineering, Indiana University, Bloomington, IN 47408; ^e^ Center for Digital Humanities & Computational Social Sciences, Korea Advanced Institute of Science and Technology, Daejeon 34141, Republic of Korea; ^f^ School of AI Convergence, Soongsil University, Seoul 06978, Republic of Korea; ^g^ Data Science for Humanity Group, Max Planck Institute for Security and Privacy, Bochum 44799, Germany

**Keywords:** art history, contextual information, multimodal AI, cultural evolution, computational humanities

## Abstract

The rise of multimodal generative AI transforms the intersection of technology and art, offering richer insights into large-scale artworks. While significant research has focused on their creative potential, their ability to represent artworks in latent spaces remains underexamined. We use generative AI, specifically Stable Diffusion, to analyze 500 y of Western paintings by extracting two types of latent information with the model: formal aspects (e.g., colors) and contextual aspects (e.g., subjects). Our findings reveal that contextual information exhibits stronger vector alignment and orientation with conventional artistic periods, styles, and individual artists than formal elements. Also, we show how artistic expression aligns with historical shifts using contextual keywords extracted from paintings. Our generative experiment, infusing prospective contexts into historical artworks, validates this vector alignment and orientation by synthesizing artworks consistent with the stylistic patterns of target periods. This study demonstrates how multimodal AI expands traditional formal analysis by integrating temporal, cultural, and historical contexts to quantify the latent structure of cultural knowledge.

## Significance

We demonstrate how multimodal AI provides quantitative insights into data-driven art history by projecting 500 y of Western paintings into CLIP’s high-dimensional semantic spaces. Unlike previous quantitative approaches focused on formal elements, our method reveals that contextual features embedded in CLIP’s pretrained representations form a continuous topological pathway aligning with artistic periods, styles, and artists. This enables quantitative analysis of content evolution, navigating semantic trajectories that mirror historical shifts like the decline of religious imagery. Image generation experiments infusing future contexts into historical works further demonstrate this vector alignment by synthesizing artworks consistent with target stylistic patterns. This framework expands conventional analysis by integrating semantic content to quantify the latent structure of cultural knowledge encoded in modern AI systems.

Understanding what artists depict in their paintings—and how they visually articulate these elements—has long been a central topic in art history. Over the centuries, this topic has been approached primarily through qualitative methods, with art historians carefully analyzing individual works and contextualizing them within broader cultural and historical narratives. However, recent technological advances have fundamentally reshaped how scholars can address such inquiries (1–13). The unprecedented availability of large-scale and high-quality digitized images of historical artworks, combined with robust analytical frameworks drawn from statistical physics (1, 4, 14, 15), information theory (6, 7, 11, 16–18), network science (19–22), and machine learning (5, 8, 23–25), has broadened the field of quantitative art research. These computational methods complement traditional analyses by offering a systematic means of rigorously testing historical hypotheses, validating or refuting prevailing insights and even uncovering previously unrecognized patterns within vast collections of visual data.

In this regard, substantial progress has already been made in quantifying what is often referred to as the “formal elements” of art—visual features such as line, shape, form, color, texture, space, and contrast—and in analyzing compositional strategies that govern how these elements are organized within artworks (26–28). Computational studies have provided a deeper understanding of aesthetic styles and trends, revealing structural patterns that extend across periods and regions. These studies have also elucidated how formal elements contribute to the perception of balance, harmony, and visual interest. As an illustrative example, early studies focused on the fundamental geometric properties of abstract paintings, e.g., Pollock’s drip paintings showing fractal patterns (1). Through further development of fractionality analysis, information-theoretic approaches have found the historical pathway of formal elements by measuring the complexity and entropy of artworks (6). Another study analyzed the temporal evolution of color contrast in paintings, showing increasing diversity across centuries and how formal elements continue to shape artistic expression (14). Studies have also examined the partition patterns in landscape paintings from a compositional perspective (7). Modern convolutional neural networks have also been used to extract visual features from paintings, revealing distinct clustering patterns among artists and styles in the principal component (PC) space (5). However, while these advances have refined our understanding of form and structure, they represent only one dimension of what art conveys: formal elements.

An equally critical but relatively underexplored aspect of art in quantitative art research is the contextual aspect. Contextual elements relate to the subjects, objects, themes, and narratives depicted in artworks, offering insights into the cultural, historical, and intellectual environments in which they were produced (10). These elements capture the essence of what artists choose to represent—whether symbolic, religious, or everyday scenes—and how these choices reflect the evolving concerns and priorities of their time. While art historians have increasingly engaged with data-driven approaches to study these contextual dimensions (29), the macroscopic study of contextual elements within quantitative aesthetics has been limited by the challenges of extracting contextual information from visual data, where variability in style, medium, and symbolic representation complicates large-scale analysis.

In this study, we operationally distinguish between formal and contextual elements as follows: formal elements refer to the visual-structural properties of artworks, specifically how they are rendered through brightness, color composition, and the structural patterns formed by the spatial organization of paintings. Meanwhile, contextual elements encompass the subjects, objects, themes, and atmospheric mood depicted in painting, that is, what artists choose to represent and the semantic content they convey. We acknowledge that this distinction is not absolute; the choice of subject matter naturally influences formal expression, and scholars may reasonably differ on where to draw these boundaries. Our primary objective is to utilize the pretrained model as a macroscope to recover and quantify the structural organization of cultural knowledge. By projecting artworks into this high dimensional latent space and comparing purely formal visual representations with semantic contextual representations, we measure the relative contributions of visual vs. semantic elements to the temporal trajectory of art history. This approach allows us to move beyond traditional formal analysis to understand how the latent structure of human culture is geometrically encoded within modern AI systems. While recent studies have successfully utilized multimodal models for specific tasks such as style classification or attribute prediction (30, 31), our framework focuses on recovering the underlying structural geometry to characterize the latent space as a continuous and navigable manifold. Thus, throughout this paper, unless otherwise specified, we employ this operational framework to distinguish formal from contextual aspects.

Recent developments in multimodal AI offer a promising pathway for overcoming these challenges (32–34). By integrating information from multiple modalities—such as images and textual descriptions—multimodal AI models can learn latent vector representations that encode not only the formal properties of an artwork but also its contextual elements. These models provide a unified framework for analyzing art at both the structural and thematic levels by bridging the gap between visual representation and semantic and contextual information.

In this study, we explored the representation of visual art within multimodal AI’s latent spaces, with a particular focus on their ability to capture the temporal evolution of art. Specifically, we examined whether these latent spaces, enriched with multimodal information, can capture chronological distinctions with greater resolution than those derived solely from formal elements. We pose the following two research questions. RQ1: Do context-enriched representations of artworks exhibit stronger alignment with the historical trajectory of art than formal representations, as predicted by the contextualist perspective? RQ2: Is the latent space of art history within a general-purpose multimodal AI model encoded as a continuous, geometrically coherent manifold that aligns with historical progression, or does it exist as fragmented, disconnected clusters? To address this, we propose a comparative framework that evaluates the relative efficacy of formal and contextual latent spaces in characterizing temporal trends in visual art. Through this analysis, we sought to demonstrate the unique potential of multimodal AI in revealing previously inaccessible dimensions of artistic change, highlighting the value of quantitative approaches that are jointly aesthetic and context driven in advancing our understanding of art history’s temporal dynamics and cultural significance.

Specifically, we used the stable diffusion model (SDM) to analyze 500 y of Western paintings (35, 36), where images are mapped onto latent vectors through its built-in autoencoder and CLIP, along with 72,447 paintings consisting of 2,354 painters and 128 conventional style periods. The autoencoder compresses high-dimensional (n=786,432) images onto a low-dimensional (n=16,384) latent space while preserving essential visual information, while CLIP simultaneously encodes visual and contextual features again in a low-dimensional space (n=1,024). We generated two distinct representation vectors for each painting: *A-vectors* from the autoencoder and *C-vectors* from CLIP. Note that A-vectors primarily capture formal visual properties, as the autoencoder is optimized purely for pixel-level reconstruction, whereas C-vectors encode both formal and contextual properties, with a primary emphasis on semantic and contextual content (37, 38). Our comparative analysis between two vectors highlights the importance of contextual elements captured by C-vectors, demonstrating their superior discriminative power among periods, artistic styles, and artists compared to A-vectors.

We then extract seminal keywords from paintings using modern language models. Using these contextual keywords, we traced changes in the motifs and subjects of Western art history and found patterns suggesting associations between artistic evolution and broader societal and technical transitions. We also attempted to reconstruct the evolution of art history through temporal diffusion experiments, in which we infused contextual elements extracted from subsequent centuries into existing paintings. Our findings provide quantitative evidence that contextual features captured by AI models correlate with temporal patterns in artistic expression, potentially reflecting broader societal changes. This approach offers a method for analyzing paintings by integrating formal and contextual dimensions and demonstrates the value of AI in uncovering deeper contextual insights beyond traditional image-based analyses.

## Results

### Time Predictability of Latent Vectors.

**Fig. 1.**
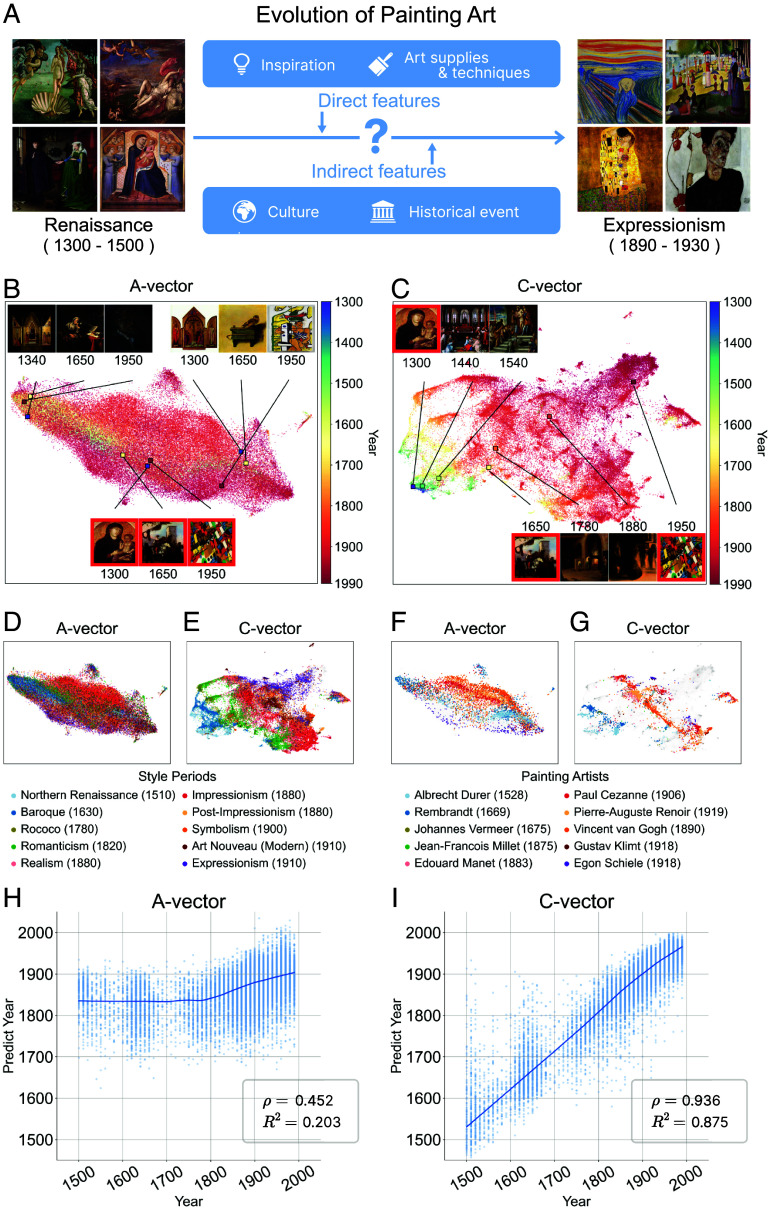
Understanding the evolution of western paintings with image embedding. (*A*) The evolution of paintings has paralleled human evolution through historical events, technological advancements, and cultural developments. (*B*–*G*) A two-dimensional (2D) projection of 72,447 Western paintings was obtained using uniform manifold approximation and projection (UMAP) with the encoded vectors (39), where each dot represents a painting. To emphasize the importance of contextual content in Western paintings, we encoded the paintings using an (*B*, *D*, and *F*) autoencoder (A-vector) and (*C*, *E*, and *G*) CLIP (C-vector) (40, 41). Note that dot colors indicate painting years. A-vectors show mixed distribution, making it difficult to distinguish painting years (*B*), while C-vectors effectively differentiate them (*C*). For instance, red-bordered paintings sampled from different periods are clustered in the center of the A-vector space but are well separated in the C-vector space. This pattern holds for the 10 most frequent conventional style periods (*D* and *E*) and 10 seminal artists (*F* and *G*). The year displayed next to the artist name is their death year, whereas those next to the conventional style period is the most frequent painting year. (*H* ans *I*) To test the expressibility of each vector, we train 100 regression models using XGBoost (42) with different train-test splits to predict painting years from embedded vectors (*Materials and Methods*). Here, solid lines are drawn with locally weighted regression (43). The results shown represent the best-performing model based on C-vector’s Pearson correlation (see *SI Appendix*, Fig. S19 for the best A-vector result). A-vectors demonstrate limited predictability (mean $R^{2}=0.202\;\pm \;0.00439$, mean Pearson ρ=0.450±0.00517), while C-vectors show remarkably high predictability (mean $R^{2}=0.869\;\pm \;0.00303$, mean Pearson ρ=0.932±0.00162), with consistently small variation across all 100 models.

Western painting is conventionally categorized by artistic movements with more or less distinctive characteristics. Understanding these characteristics benefits from examining both formal elements and contextual dimensions. The evolution of artistic movements can be understood in relation to each era’s cultural, social, and philosophical contexts (Fig. 1*A*). For instance, the development of new painting tools and steam-powered transportation facilitated the emergence of landscape paintings, whereas the Industrial Revolution influenced the rise of impressionistic works (10). Building on this foundation, we examined how to capture the evolution of paintings by extracting both formal and contextual information.

One advantage of encoders in AI models is that they learn important features while reducing the dimension of the original inputs. We employed an autoencoder and CLIP within the SDM to embed painting images into two distinct latent spaces, producing A- and C-vectors that encode formal and contextual features, respectively (*Materials and Methods*). The uniform manifold approximation and projection (UMAP)—a dimensionality reduction method that groups neighboring paintings within the manifold structure of a high-dimensional space (39)—indicated that A- and C-vectors form distinct spatial distributions. The UMAP of the A-vectors exhibited a clustered pattern based on visual characteristics but did not show distinct clusters by painting year (Fig. 1*B* and *SI Appendix*, Fig. S5). In contrast, the UMAP of the C-vectors displayed a progressive trend with time (Fig. 1*C* and *SI Appendix*, Fig. S5), from 14th-century paintings occupying the bottom left to 20th-century paintings positioned in the upper right. This continuous progression from the bottom left to upper right reveals a chronological pattern across centuries. Notably, paintings from the 19th and 20th centuries were widely distributed in the C-vectors (Fig. 1*C*), but they remained distinct without mixing with other periods. In the A-vectors, however, paintings from these same periods showed no such temporal separation, suggesting that A-vectors were less suitable than C-vectors for capturing temporal characteristics in our UMAP visual clustering analysis.

One may argue that this can be attributed to the larger number of paintings in recent periods (*SI Appendix*, Fig. S1), yet we consistently observed limited expressibility of the UMAP-clustered A-vectors. For example, although we can observe some clustered structures in the UMAP-clustered A-vectors when grouping paintings by conventional style periods and artists, the degree of clustering in these groups is considerably weaker than that in C-vectors (Fig. 1 *D* and *F* and *SI Appendix*, Figs. S6 and S7). Despite the significant divergence in the number of paintings across different artists and conventional style periods (*SI Appendix*, Figs. S2 and S3), the UMAP of the C-vectors also demonstrated well-bounded clustering according to conventional style periods and painting artists (Fig. 1 *E* and *G* and *SI Appendix*, Figs. S6 and S7). This is further confirmed by the distances between paintings in the A- and C-vectors (*SI Appendix*, Fig. S11). C-vectors showed a significantly larger separation between different authors (mean distance: 1.138 vs. 0.959 for different and same authors, respectively, with 18.7% difference) than A-vectors (mean distance: 153.010 vs. 143.753 for different and same authors, respectively, with 6.4% difference). This better discriminative power was also confirmed by the Kolmogorov–Smirnov (KS) test, where C-vectors showed a substantially higher KS statistic between the same and different author distributions (0.614) compared to A-vectors (0.206). C-vectors also exhibited more consistent measurements across different styles (KS statistic: 0.330 vs. 0.053 for C- and A-vectors, respectively; mean distance: 1.122 vs. 1.039 for C-vectors; 149.150 vs. 146.631 for A-vectors).

We also observed that related artists and styles clustered close to each other. For example, the paintings of Gustav Klimt and Egon Schiele are closely positioned in the C-vector space, reflecting their historical and stylistic connections (Fig. 1*G* and *SI Appendix*, Fig. S7). The two artists lived in Vienna in the early 20th century and profoundly influenced each other’s works. However, the paintings of Pierre-Auguste Renoir are separate from these two artists’ works in Fig. 1*G*, reflecting the French impressionist’s distinct artistic approach and geographical separation from the Viennese artists. In contrast, A-vectors organized these artists differently, with Klimt and Renoir located together and Schiele and Klimt separated from each other (Fig. 1*F* and *SI Appendix*, Fig. S7), highlighting how formal features can group paintings differently than contextual features. Note that we obtained similar patterns with another dimensionality reduction method (t-SNE; see *SI Appendix*, Figs. S8–S10), confirming the consistency of these patterns across different dimensionality reduction-based visualization methods.

To quantify the temporal resolution of the A- and C-vectors, we evaluated their capacity to predict the historical period of artworks. We employed the XGBoost regression model, which is widely used in machine learning tasks (42). For each vector type, we trained separate models using 70% of the original paintings as training data, with their respective latent vectors as input features to predict the year of creation. We then assessed the predictability of the models using the remaining 30% of paintings (see *Materials and Methods* for further details). The locally weighted regression line (43) suggests that the A-vector model predominantly misclassified early paintings (from the 16th to 18th centuries) as 19th-century works (mean $R^{2}=0.202\;\pm \;0.00439$; Fig. 1*H* and *SI Appendix*, Table S6). The model also predicted numerous 20th-century paintings as 19th-century works. Meanwhile, the C-vector model consistently showed more accurate creation year predictions across 100 models with different train-test splits (mean $R^{2}=0.869\pm 0.00303$; Fig. 1*I* and *SI Appendix*, Table S6). In addition, the C-vector model showed better classification metrics (e.g., F1 score and accuracy) across all levels of error tolerance (*SI Appendix*, Fig. S17). In short, the C-vectors exhibited superior predictability for artwork creation year compared to the A-vectors, both in overall accuracy and in avoiding systematic misclassification of early and late period paintings. We additionally conducted an artist-based train/test split to verify the robustness of our results against potential artist confounding effects, where a model may learn the style of a specific artist rather than genuine temporal patterns, confirming results consistent with our original findings (*SI Appendix*, Fig. S23). The C-vector model also showed better classification performance when predicting 10 art movements and 10 artists selected in Fig. 1*D*–*G*, with superior metrics (e.g., F1 score and accuracy; see *SI Appendix*, Fig. S20 and Table S7). This finding aligns with CLIP’s intended capability to capture semantic relationships between images and text, demonstrating that these pretrained representations from foundation models, even when trained with a general web-crawled dataset (specifically, a subset of LAION-5B), effectively transfer to art historical analysis.

One may ask whether these results simply reflect the memorization of training data by CLIP. To address this concern, we reproduced the analysis using SynthCLIP (44), a CLIP model trained exclusively on synthetically generated image-text pairs, that has never been exposed to any real paintings. As shown in *SI Appendix*, Fig. S22, the UMAP projection of SynthCLIP vectors reveals a temporal structure in the embedding space, and the regression model obtains $R^{2}=0.507$ and Pearson ρ=0.712, substantially higher than the A-vector baseline. Note that SynthCLIP achieves lower performance than the original CLIP (ρ=0.712 vs. 0.932), which could be due to the general inferiority of the synthetic-only model or evidence of memorization in CLIP. However, the significantly higher performance of SynthCLIP than the A-vector baseline suggests that the temporal structure we observe is not merely an artifact of training data memorization, but reflects a genuine structural property of multimodal representations: even without exposure to real paintings, the embedding space organizes artworks into a continuous, temporally coherent structure.

### Unveiling Latent Information Encoded in Vectors.

In the previous section, we observed that C-vectors exhibit greater predictive power than A-vectors in terms of temporal classification, demonstrating higher accuracy in predicting painting years and distinguishing artistic periods/artists. Thus, a natural question arises: what makes C-vectors more expressible than A-vectors? To answer this question, we conducted an in-depth analysis of the encoded information, particularly for paintings, within the A- and C-vectors to unveil the factors that make C-vectors more interpretable. Because both the autoencoder and CLIP models were trained using general image datasets, we first performed principal component analysis (PCA) on the A- and C-vectors to extract the key features of paintings. We then investigated how paintings vary in style and context along each PC axis to interpret what each axis encodes.

**Fig. 2.**
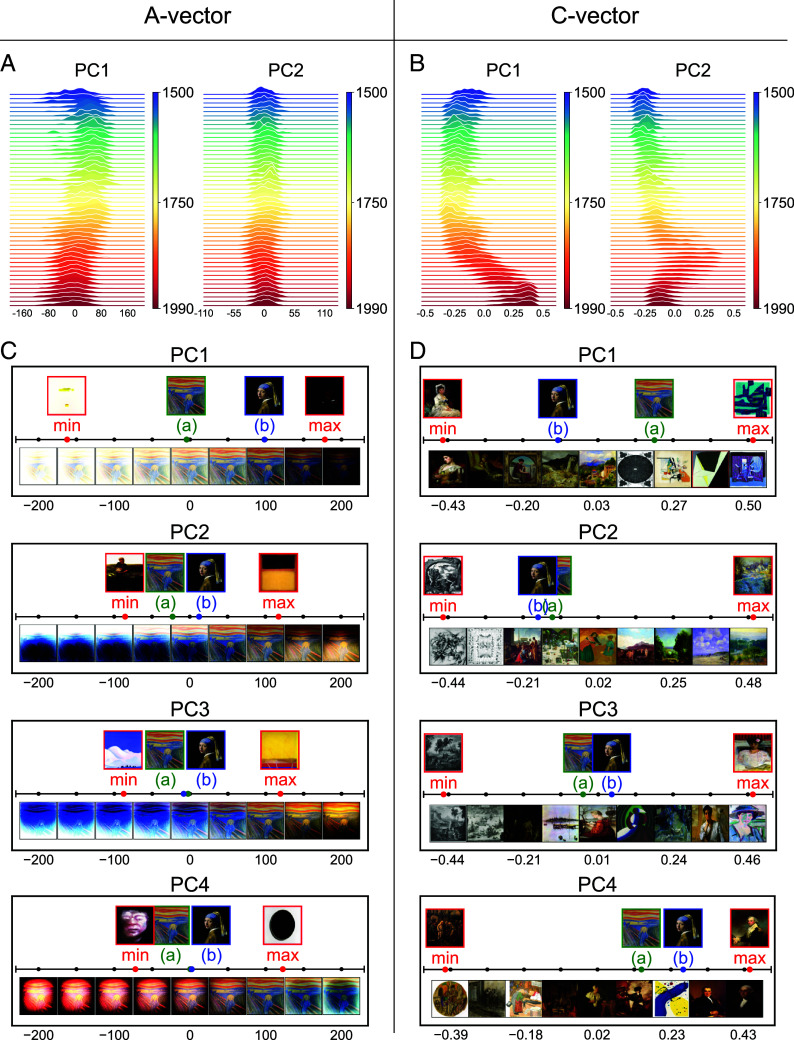
PCA reveals latent information encoded in embedded vectors. To explore the expressibility gap between A- and C-vectors, we conducted PCA to extract principal information from each vector representation. The first two components of each vector space revealed that (*A*), A-vectors show minimal variation, while (*B*), C-vectors demonstrate substantial temporal differentiation. For (*A*) and (*B*), the x-axis is bounded by the maximum and minimum projected values of paintings on the PC. (*C*) We obtained modified A-vectors of Munch’s “The Scream” using vector analogy: $v_{\text{new}}=v_{\text{original}}+d\cdot PC_{\text{i}}$, where $v_{\text{original}}$ represents the embedded vector of “The Scream” and $PC_{\text{i}}$ denotes the normalized i-th PC vector. Images were then generated using the SDM’s autoencoder with these modified vectors (35). The resulting images show that the first four PCs of the A-vector primarily represent visual composition elements: 1) brightness, 2) vertical brightness composition, 3) hue (blue to yellow), and 4) highlight distribution. The original vector of “The Scream” is placed near the zero point, while Vermeer’s “Girl with a Pearl Earring” has a high PC1 value. (*D*) For comparison, we retrieved paintings based on t heir C-vector’s projected values and distances on each PC (see *Materials and Methods* for detailed selection criteria), as CLIP’s encoder-only architecture lacks image generation ability. We observed that images mainly vary in context along the PCs, as exemplified by transitioning from portraits to abstract compositions in PC1.

We first projected paintings onto the PCs by computing the dot product between the latent vectors of paintings and each PC. The distributions of the projected values on the first- and second-largest PCs (PC1 and PC2, respectively) of the A-vectors initially appeared to show little evidence of temporal patterns (Fig. 2*A*). We noticed subtle temporal variations, with a modest increase during 1500 to 1550, followed by a plateau period between 1550 and 1750, before a gradual decline throughout 1750 to 1990 for PC1; meanwhile, the PC2 distribution remained temporally consistent. We observed a similar temporal stability across the remaining PC axes of the A-vector (*SI Appendix*, Fig. S13). It should be noted that the eigenvalues (i.e., explained variance ratios) of the PCs for both the A- and C-vectors exhibited steeply declining distributions (*SI Appendix*, Fig. S12); the largest PCs (corresponding to the largest eigenvalues) largely explain the vital information that the vectors encode for paintings. As PC1 has a high explained variance ratio (0.190), which is almost six times that of PC2 (0.034), PC1 shows the significant expressibility of paintings for the A-vector. Therefore, although the PCs of the A-vector effectively characterize certain aspects of paintings, they lack a clear temporal correspondence.

In contrast, the C-vector demonstrated more drastic temporal variations in PCs over time (Fig. 2*B*). On the PC1 axis of the C-vector, the projected values showed a rapid increase from the late 1800s and then saturated from 1970. PC2 peaked in the late 1800s and then declined, displaying an inverse temporal trajectory compared to PC1. Similar distinct temporal patterns were observed across the remaining PC axes of the C-vector (*SI Appendix*, Fig. S13). Note that these two PCs maintained relatively low explained variance ratios (0.063 and 0.050), and the explained variance in the C-vectors decreased more gradually than that of A-vectors (*SI Appendix*, Fig. S12), suggesting that the information in the C-vectors was more evenly distributed across the principal dimensions of the artwork. In short, these temporal patterns indicated that the features captured by the C-vectors exhibited stronger temporal sensitivity (*SI Appendix*, Fig. S13).

Next, we investigated the aspects of paintings encoded by each PC by modifying the PC values of paintings for both vector types. The autoencoder comprised both encoder and decoder models, enabling us to generate images directly from the modified A-vectors. In contrast, CLIP is an encoder-only model, which prevented the generation of images directly from the C-vectors. Consequently, we employed different analytical strategies for each vector type. For the A-vectors, we generated new latent vectors by manipulating the paintings along the PC directions as follows:

[1] $$\begin{array}{c} v_{\text{new}}=v_{\text{original}}+d\cdot PC_{i},: \end{array}$$

where $v_{\text{original}}$ is the A-vector, $PC_{i}$ is the **i**-th PC, and d is a scalar parameter that adjusts the position along the PC axis ranging in (−200, 200). We then generated images of $v_{\text{new}}$ using the decoder part of the autoencoder. This method allows us to monitor how a painting changes as we adjust its position along the PC axis, revealing which aspects of the artwork each PC represents. As an illustrative example, we show modified versions of Edvard Munch’s “The Scream” by adjusting $v_{\text{original}}$ in Fig. 2*C*. For the C-vector, because there is no decoding method for $v_{\text{new}}$, we found the painting closest to each point along the PC axis (see *Materials and Methods* for details).

Then, what does each PC axis represent? We found that each PC of the A-vectors captures specific visual elements, such as brightness, hue, and composition (Fig. 2*C*). The PC1 axis predominantly encodes the overall brightness, where paintings with brighter tones have a negative value and darker compositions have a positive value. These brightness variations manifest more intensely at the peripheral boundaries of the paintings than in their central regions, corresponding to the common artistic practice in which foreground subjects are spotlighted more than background elements (see PC4). As an illustrative example, Johannes Vermeer’s “Girl with a Pearl Earring,” which features the main subject (girl) centered against a dark background, is positioned at ∼98.27 on the PC1 axis, whereas Edvard Munch’s “The Scream” occupies a more neutral value (∼−4.53). PC2 captured the horizontal compositional elements of the paintings. The minimal abstract work “No.9 (Dark over Light Earth)” by Mark Rothko, featuring black blocks positioned upon yellow, is located at the maximum value (∼117.45) of the PC2 axis, whereas at the opposite extreme (minimum, ∼−85.48), “Portrait Of Orca Bates 1989” by Jamie Wyeth, displays a composition with bright areas contrasted by darker elements at the bottom. PC3 represent colors—blue to orange. PC4 also emphasize different compositions and highlight patterns. In summary, the A-vector apparently mainly encodes the “formal element” of artworks, which has been a dominant subject of study in quantitative aesthetics (7, 14, 26–28).

In contrast to the A-vector, the PC axes of the C-vector primarily captured the semantic and contextual elements of paintings, e.g., subject matter, historical context, and stylistic movements (Fig. 2*D*). For instance, on the PC1 axis, paintings featuring human subjects and portraits occupied the leftmost negative values (minimum PC1), whereas more geometric or abstract compositions had the rightmost positive values (maximum PC1). This result is consistent with our observations of the PC1 projected values in Fig. 2*B*, showing a rapid increase from the late 1800s to the late 1900s. PC1 thus aligns with the structural transition toward abstract art during this period, quantifying the geometric shift in the latent space that encodes the evolution of artistic representation. The PC2 axis distinguishes between different subject domains, separating human figures (negative values) from landscape paintings (positive values). For example, both “The Scream” and “Girl with a Pearl Earring” occupy negative values on the PC2 axis. As we observed increasing PC2 values from the late 1700s, with a peak in the late 1800s (Fig. 2*B*), this pattern aligns with the historical proliferation of landscape painting, driven by technological advances, e.g., steam locomotives and tube pigments (45, 46). PC3 introduces further nuance, differentiating between simple portraits and more complex compositional scenes in which human subjects appear within elaborate backgrounds. These distinctions suggest that C-vectors encode not only visual objects but also deeper contextual relationships within artistic traditions. Some PCs capture more complex variations that humans cannot easily capture; e.g., PC4 does not follow a simple regularity but reflects a more intricate structure, capturing hidden context gradually transitioned by time that even untrained humans hardly perceive (*SI Appendix*, Fig. S13). This demonstrates the further potential of the AI model in art history studies. Despite this complexity, images with smaller PC4 values generally display more realistic features than those with larger PC4 values, indicating that this dimension may capture the degree of artistic realism vs. stylization.

These findings reveal the differences in the information embedded in the A- and C-vectors and demonstrate that each vector characterizes distinct temporal changes in the features of artworks. The A-vector principally captures features of the structure of the paintings, i.e., formal elements, whereas the C-vector principally encodes information about the objects, i.e., contextual elements. As shown in Fig. 1, the A-vector has limited expressibility for the creation year, artist, and conventional style period of paintings. This observation suggests that for understanding the historical evolution of art, contextual information captured by C-vectors provides critical complementary insights beyond formal elements alone. C-vectors effectively capture this contextual dimension, allowing us to detect distinctions in the creation year, artist, and style of paintings. In other words, incorporating contextual information with the social and historical backgrounds of paintings is essential for a deeper understanding of the evolution of art.

### Contextual Evolution of Paintings.

In the previous section, we demonstrated that C-vectors effectively capture contextual information in paintings and better reflect temporal changes in artworks. This finding naturally leads us to question whether the content and contextual information depicted in paintings genuinely reflect societal changes. The literature supports the idea that social change can lead to the emergence of new artistic styles. For example, scholars have identified how air pollution in the 19th century contributed to the emergence of impressionism (10). Combined with our findings, we assume that extracting human-understandable contexts encoded in C-vectors from paintings should enable us to trace social changes. Therefore, we designed an analysis that extracted contextual keywords from paintings and analyzed their temporal changes as implicit in the AI model and by extension as recorded in the original training data.

We assume that a text prompt represents an image most accurately among alternatives if the original image can be regenerated from the prompt in the most similar form. Thus, we inferred the generative prompts by CLIP Interrogator (47), combining the CLIP (41) and BLIP (48) models, in which the prompt reproduces the original image as much as the SDM can (*Materials and Methods*). We then separated each prompt into (1-g) keywords using delimiters (e.g., spaces and commas) and calculated their normalized frequencies, where the frequency of each word was divided by the total sum of all word frequencies within its respective decade.

**Fig. 3.**
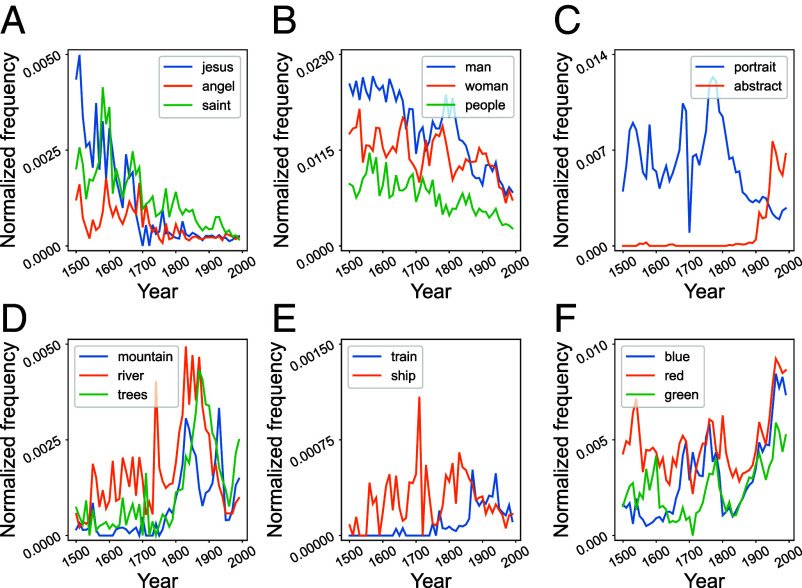
Generative prompt outlines temporal evolution of western art. (*A*) Religious words show decreasing trends over time. (*B*) Similarly, human subject descriptors also gradually decreased. (*C*) Comparative analysis of artistic style descriptors *abstract* and *portrait*, highlighting the transition in representational approaches. (*D*) Natural features abruptly increased around the 1800s, likely reflecting the development of tube colors and steam locomotive trains enhancing the mobility of painters. (*E*) We also observed notable changes in transportation-related keywords. For example, the term *train* steeply increased following the invention of steam locomotives in the 1800s. (*F*) The rise of simple color words indicates an evolution toward an abstract style in the early 1900s. These findings highlight how multimodal AI models can effectively illustrate shifts in artistic expression and content in response to technological, societal, and stylistic changes throughout history.

Changes in representative keywords over time revealed intriguing patterns in the painting content. We first detected a substantial decline in religious keywords, such as *jesus*, *angel*, and *saint*, which became rare after 1700 (Fig. 3*A*). This pattern reflects society’s gradual disengagement from religion over time. Meanwhile, human-related keywords (*man*, *woman*, and *people*) also decreased yet maintained a considerable portion (Fig. 3*B*). In this regard, our comparative analysis of artistic style descriptors revealed a pronounced increase in the term *abstract*, coupled with a corresponding decline in *portrait*, which primarily depicts humans. This trend emphasizes the fundamental transition in representational practices throughout art history as implicit in the AI model (Fig. 3*C*; see Fig. 2 *B* and *D* also for the increment of PC1, showing an increasing trend of abstract arts). We observed a significant increase in keywords related to landscape painting, such as *mountain*, *river*, and *trees*, starting from the late 1700s and peaking in the late 1800s (Fig. 3*D*); this trend corresponds with technological advances, such as steam locomotives and tube pigments, as reflected in our PCA results (see the steep increase in PC2 in Fig. 2 *B* and *D*). We further confirmed this pattern through the increment of the keyword *train* (Fig. 3*E*). Primary colors also increased significantly since the 1800s, coinciding with the emergence and rise of abstract styles (Fig. 3*F*). These temporal patterns remained robust after excluding style-specific keywords, which appeared in less than 0.4% of total frequencies (*SI Appendix*, Fig. S21). We also identified the most increased and decreased keywords over the five centuries through regression analysis (see *SI Appendix*, Tables S1 and S2, respectively, along with *SI Appendix*, Fig. S14). This analysis validates the results shown in Fig. 3. For instance, *abstract* ranked as the 3rd most increased keyword, whereas color-related terms ranked highly (e.g., *blue* as 2nd, *white* as 4th, *yellow* as 5th, *color* as 10th, *green* as 12th, and *red* as 17th). On the other side, human-related terms (e.g., *man*, *people*, *woman*, and *portrait*) and religious terms (e.g., *jesus*, *saint*, and *angels*) ranked as the most decreased keywords.

One may ask whether these temporal patterns in visual representations are truly influenced by societal changes. While directly establishing a causal mechanism is challenging (49), we address this question alternatively by comparing our findings with an independent historical source: selected keywords with Google Books N-gram frequencies from 1,500 to 2,000 (*SI Appendix*, Fig. S18) (50). The analysis revealed meaningful alignments: invented objects like *ship* and *train* showed similar temporal patterns across both media, while natural subjects such as *mountain* and *trees* exhibited strong positive correlations (Pearson ρ=0.385 to 0.890). On the other hand, subjects like *jesus* showed negative correlation (ρ=−0.192), revealing medium-specific patterns where religious imagery declined in paintings while remaining prevalent in books around the 1700s.

In summary, we observed notable evidence that social changes, including the evolution of painting tools, technological advances, and social events are encoded into AI models, capturing the formation and development of paintings. The emergence of structured patterns from the heterogeneous dataset (i.e., LAION-5B) demonstrates a robust latent semantic pathway. Notably, high-quality artistic images constitute only 0.16% of the training data (LAION-art subset: 8M images), which includes diverse media types alongside paintings. Despite the overwhelming contemporary noise in the training data, Western painting’s distinct trajectory reveals a geometrically organized representation of historical evolution encoded within the model.

### Future Contextual Information Navigates Forthcoming Paintings.

Our previous findings revealed that contextual information extracted from paintings is closely related to societal changes and that this information can successfully regenerate images similar to the original ones (Fig. 3 and *SI Appendix*, Fig. S4). The robust expressibility of C-vectors also suggests their potential utility beyond reconstruction. Because contextual features effectively encode temporal progression in artistic development, we hypothesized that manipulating these contextual features of paintings might allow us to systematically generate images that align with different temporal periods. This relationship led us to the interesting question: can contextual information from later periods, when combined with earlier artworks, generate images that are consistently identified as characteristic of those later periods? Unfortunately, we cannot determine whether the generated artistic style truly represents the future. Instead, we examined whether our approach could systematically generate images that align with the expected characteristics of subsequent periods. We investigated whether images generated from paintings from a specific period, infused with contextual information from subsequent periods, were predicted by our temporal regression model as belonging to those later periods.

To explore this possibility, we designed the following generative test. First, we selected the 77 representative keywords for each century based on their TF-IDF values, where an artwork corresponds to a document (see Materials and Methods for detailed selection criteria; see *SI Appendix*, Table S5 also for the full selected keywords list). These keywords were derived from paintings from each century using the same text extraction method described in the previous section. We then generated space-separated prompts for each century (see *Materials and Methods* for details; testing of alternative comma-separated prompts is also presented in *SI Appendix*, Fig. S16). Second, we randomly sampled 500 paintings from each century. Third, for paintings from the *t*-th century, we generated paintings with the keywords of the (t+1)-th century using the SDM (35). For the comparative null model, we also generated painting images from the same t-th century images using the same SDM but without providing any keywords. We refer to the former (with keywords) as *future-directed paintings* and the latter (without keywords) as *random diffusion paintings*. The SDM generates an image by guiding the diffusion direction based on the text input. Therefore, we assume that the keywords of the (t+1)-th century guide the diffusion process toward the future from the original image, where an image without any text input is assumed to be randomly directed. We repeated this process by varying the step parameter to modify the degree of perturbation. This process allowed us to observe both the evolution of paintings and degree of transformation influenced by the text input (*Materials and Methods*).

**Fig. 4.**
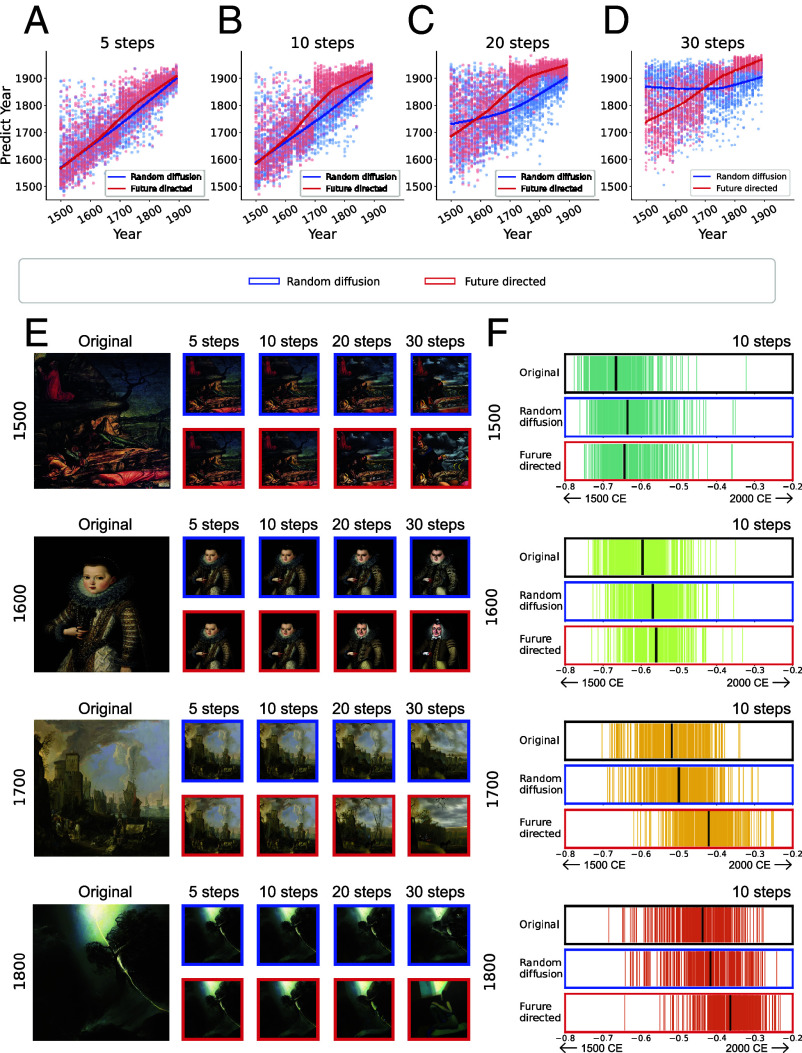
Replicating evolutionary trajectories of western paintings. To verify our findings on the role of context in understanding the evolution of art, we designed a simple generative experiment with an image-to-image diffusion model using different prompt guidance: one used a null prompt (random-diffusion), while the other used representative keywords of the next century, e.g., keywords of the 1800s for the paintings of the 1700s (future-directed; see *Materials and Methods*). (*A*–*D*) Using the generated images, we first estimated the painting year for each using the best C-vector regression model shown in Fig. 1, which showed that the future-directed images (red lines) tend to be predicted consistently ≃100 y ahead of their source painting, demonstrating a systematic temporal shift in artistic characteristics. In contrast, the random-diffused images (blue lines) showed a distinctive convergence toward ∼1900 as the diffusion steps increased. Here, solid lines are drawn with locally weighted regression (43), and steps control the relative noise level, where larger steps reduce the consistency with the original image by applying stronger noise (*Materials and Methods*). (*E*) Sampled images for each period, showing that diffusion steps do not alter the large formal elements of paintings for both random-diffused (blue-boxed) and future-directed (red-boxed) images. (*F*) Spectrum of the paintings. The color represents the original period of the painting, and the position reflects the projected value of C-vector onto the temporal axis $v_{1500s\to 1900s}$ for original (black-boxed), random-diffused (blue-boxed), and future-directed (red-boxed) images at step 1 (*Materials and Methods*). For all panels, we randomly sampled 500 images from each period to reduce interpretation bias rooted in data display imbalance.

The results show that future-directed paintings were more accurately classified as works from the subsequent century relative to their original creation time compared to random diffusion. Notably, as the number of diffusion steps increased, the images generated by the random diffusion model tended to be classified as paintings from the 20th century, where modern abstract art became more prevalent. One might wonder whether this reflects characteristics of the SDM training data (LAION-5B). However, we attribute this classification tendency to the use of randomness in modern artistic practices, as exemplified by highly experimental artists such as Jackson Pollock (4). We can reasonably infer that the model has difficulty differentiating between pure random patterns and intentional artistic randomness. Our investigation confirmed this limitation; when we input pure white noise patterns into the year prediction model, they consistently predicted years around the mid-20th century (*SI Appendix*, Fig. S15). By contrast, the temporally future-directed images showed a plausible temporal progression (Fig. 4*A*–*D*). Varying diffusion steps produced a progressive shift along the temporal axis, demonstrating that the latent space is organized as a continuous, navigable pathway rather than discrete jumps. This structural order emerges despite the inherent heterogeneity of the training data. However, one should caution that potential data imbalance in the training dataset (i.e., LAION-5B) cannot be completely excluded.

An interesting observation from the generated images—both future-directed and random-diffusion—is that they closely resemble the original paintings in terms of their formal aspects (Fig. 4*E*). The paintings retain the same outlines, colors, and compositions, indicating that their formal characteristics remain largely unchanged. However, upon closer inspection, subtle differences in content emerged. For instance, a sample portrait painting from the 1600s in Fig. 4*E* underwent a dramatic transformation in the hairstyle and clothing of the portrait subject as the diffusion steps increased. At 30 steps, the subject appeared to adopt a silver-colored wig, which corresponds to the wig vogue in the 18th century (51); note that the term “wig” is also the top keyword of the 18th century (*SI Appendix*, Table S5). Additionally, a sample painting from the 1800s transformed from a tree into an abstract human figure, reminiscent of surrealist art. These findings suggest that contextual information, represented by keywords extracted from certain periods, guides the diffusion process to generate images whose contextual features align with the characteristics of those later periods, while preserving their formal attributes.

Finally, from an alternative perspective, we confirmed that future-directed paintings exhibited a stronger tendency to move toward the future. We calculated the temporal axis $v_{1500s\to 1900s}$ as the difference between the average C-vectors of all paintings from the 1500s and those from the 1900s (*Materials and Methods*). When projecting paintings onto the vector axis captur- ing the temporal variation from 1500 to 1900 in the C-vector space, future-directed paintings consistently shifted toward higher average values compared to random-diffusion paintings and original paintings, except for those from 1500, as shown in Fig. 4*F*. Random-diffusion paintings also exhibited higher average values than original paintings, although the difference was marginal. Note that the results are robust regardless of the prompt format (*Materials and Methods* and *SI Appendix*, Fig. S16).

In summary, we confirmed that future paintings can be induced through experiments that infuse future context into a painting without altering the formal elements. This demonstrates that contextual features, as captured by multimodal AI models, play a crucial role in determining temporal characteristics of artworks. These findings suggest that the contextual information encoded in paintings correlates with their temporal positioning, consistent with the hypothesis that artistic characteristics are influenced by their broader societal context.

## Discussion

In this study, we investigated how multimodal AI can enhance our understanding of art history by analyzing the contextual elements in paintings. Although these contextual elements are widely recognized as key drivers of artistic evolution (52, 53), they have remained largely unexplored in data-driven studies owing to methodological challenges. Recent advances in AI, particularly through the vision-to-text multimodal approach, have enabled the systematic analysis of painting contexts, as exemplified by the CLIP-based C-vector extraction in this study. Our multimodal AI approach, combined with a massive art dataset (36), provided an opportunity to quantitatively explore the evolution of human creativity by connecting traditional art historical approaches with data-driven methods.

Our findings reveal a fundamental difference between the two latent spaces: a-vectors, optimized for generative image reproduction tasks, capture formal visual features that enable realistic image synthesis, while C-vectors encode contextual relationships that align more closely with art-historical understanding. This distinction aligns with recent findings on the differential coverage of aesthetic (formal) vs. semantic (contextual) dimensions in various image embedding approaches (54) and might suggest the cognitive difference between the skills required for creating art and those needed for art-historical scholarship. The superior temporal expressibility of C-vectors compared with A-vectors highlights the significant role of transformations in contextual content in addition to purely aesthetic transformation in shaping the evolution of art (Figs. 1 and 2). The distinctive stylistic signatures of individual artists and conventional style periods are reflected more precisely in C-vectors than in A-vectors. We also found remarkable temporal continuity of C-vectors on UMAP, showing linear trajectories across periods, which indicates that artistic contexts can be imagined to evolve progressively.

Additionally, our procedure extends culturomics (50) to the visual arts domain by combining C-vectors and generative AI. We observed that the frequency of contextual elements in paintings suggests associations with societal changes, demonstrating that computational analysis of art can quantitatively capture cultural evolution (Fig. 3) (at least) as inherent in large image collections and trained machine learning models. This approach contributes to the broader field of cultural analysis, which has moved beyond early text-based methods that were limited by biases in data sources and simplistic frequency measures (55). While our visual dataset has its own biases, such as its reliance on canonical and highly acclaimed artworks, it offers a complementary layer within a wider cultural analysis. Our work also aligns with the growing emphasis in cultural analytics (56) on incorporating multiple types of data, extending beyond visual art to include music (57, 58), historical newsreels (59), and film (60). In this context, our framework, which extracts meaningful contextual information from artworks using multimodal AI, contributes to a more comprehensive understanding of the dynamic interplay between art, society, and culture.

SDM-based image generation with an infusion of future contextual information revealed that the evolution of artistic expression can be systematically modeled by modulating contextual elements without changing formal elements (Fig. 4). In other words, even when the same formal aspects remain familiar, changes in context can lead to new artistic expressions, indicating that painting remains an ever-evolving medium.

This study did have some limitations. First, our dataset primarily consisted of well-known Western artworks, which may introduce geographical and temporal biases. We indeed observed an abruptly increasing volume of artworks in recent years (*SI Appendix*, Fig. S1). Second, our filtering criteria (as described in *Materials and Methods*) may have resulted in uneven representation across different periods, styles, and artists. Our work also relied on SDM, a specific AI model with finite expressibility (e.g., 77 tokens limitation), indicating that future studies could benefit from advances in AI. Third, our operational distinction between formal and contextual elements, while practically acceptable for this analysis, involves some degree of abstraction; the boundaries between visual structure and semantic content are not absolute, and alternative categorizations may offer complementary insights. Fourth, while some artworks may have been included in the models’ training datasets, we emphasize that such internalization of cultural data is a necessary prerequisite for our structural analysis. Rather than viewing this overlap as a bias, we treat the model as a macroscope that enables us to recover and quantify the structural organization of art historical knowledge. This effect should be limited because the SDM and CLIP are general-purpose models trained on a large-scale dataset, and our SynthCLIP analysis suggests that memorization effects do not solely drive the temporal structure observed in this study. Nevertheless, since SynthCLIP was trained on images generated by Stable Diffusion, which was itself trained on real artworks, some indirect traces of artistic style may be embedded in its representations, though we expect this effect to be marginal. Our findings demonstrate that the vector alignment and orientation in the latent space reveal the geometric organization of art history as encoded within the model. Our analysis establishes the structural organization and geometric coherence of the latent space, demonstrating how artistic evolution correlates with historical progression. While our focus is on quantifying this structural foundation rather than testing causal mechanisms, this work provides the necessary prerequisite for future investigations into relationships between latent pathways and socioeconomic indicators, ensuring subsequent analyses can work with meaningful cultural patterns rather than arbitrary feature clusters.

Despite these limitations, our contextual approach provides a promising direction for data-driven art history research (29) by demonstrating its robust validity and performance. Potential extensions are required to enhance the impact of the study; for instance, analyzing the temporal evolution of specific contextual elements (e.g., human figures and landscapes) regarding their formal representations, extending the analysis to non-Western art (e.g., Korean landscape paintings *Sansu-hwa* and Japanese woodblock prints *Ukiyo-e*). Investigating connections between our identified contextual elements and documented socioeconomic changes (49), such as GDP, trade dynamics, political transitions, war, and historical events, will also be a promising future direction. By combining computational methods with art-historical expertise, such work may ultimately contribute to a richer understanding of how artistic expression evolves across cultures and historical periods.

## Materials and Methods

The ART500K dataset (36) comprises paintings collected from Google Arts & Culture (61), WikiArt (62), Web Gallery of Art (63), and other sources. The dataset provides comprehensive information on each painting, including 41,096 painters, dates from BC to the 2000s, 726 artistic styles, 114 artists’ nationalities, and other attributes.

### Data Preprocessing.

Because this study aimed to analyze the evolution of Western paintings, we preprocessed the dataset using the following steps. First, we removed paintings without image files from the ART500K dataset because our analysis was based on images. We then extracted the painting date from the Date column of the Art500K metadata. When the painting year was not explicitly described, we extracted the year from the title (painting_name column), if available. Because approximately 25% of the artworks lack a specific creation year but contain approximate values (e.g., 1846–1848, c1540, or 1420s), we grouped paintings by decade (e.g., 1846–1848 to 1840). For artworks spanning multiple decades, we selected the final year and performed decadal rounding down (e.g., 1539–1542 to 1540). This decadal grouping had limited impact on our analysis because most conventional artistic style periods typically span 10 or more years (64). In this study, we utilized these grouped values to analyze painting years, unless otherwise specified. After these preprocessing steps, paintings without year information were excluded from the analysis. We also filtered the dataset using the metadata labels in the following order: Style, Field, Genre, and Nationality. Using this order, we first removed entries containing removed keywords (*SI Appendix*, Table S3), and then retained only entries containing the selected Field keywords (*SI Appendix*, Table S4). If the artist’s name contained non-English letters, it was normalized using Python’s internal unicodedata.normalize() function.

In this study, we used the structure of the SDM 2.0 (https://github.com/Stability-AI/stablediffusion). We used the checkpoint trained to generate images of 512×512 pixels by default (512-base-ema.ckpt), with all adjustable parameters set to their default values. All images were resized to 512×512 pixels following the model specifications. We removed 3.2% of the remaining dataset where one dimension was at least twice that of the other to prevent information loss during the resizing process. In addition, we removed ∼8% of the remaining paintings with resolutions lower than 410×410 (in other words, each dimension was lower than ∼80% of 512) to maintain sufficient image quality for analysis. Finally, we restricted the data to years 1500 to 1990 because the number of available artworks before 1500 was insufficient for analysis (*SI Appendix*, Fig. S1). The final processed dataset contained 72,447 paintings, consisting of 2,354 painters and 128 conventional style periods. Note that the number of paintings in each decade group increased (*SI Appendix*, Fig. S1).

### Encoding Paintings with Latent Vectors.

We extracted two types of latent vectors: the A-vector using SDM’s autoencoder, which compresses the original image dimensions from approximately 0.8 million (512×512×3=786,432; excluding alpha channel) to 16,384 dimensions, and the C-vector using the CLIP model checkpoints from SDM2.0, with a dimension of 1,024. These complementary representations enabled us to investigate both the formal and contextual elements of artworks. The autoencoder transforms an input image *x* from RGB space into a latent representation $L_{a}=E\left(x\right)$ by training the model to replicate the input image with a bottleneck structure (65). By contrast, CLIP maps both images and text into a shared latent space, where semantically similar content is positioned closer together (41).

For Fig. 2*D*, we selected sample paintings for each projected value of each PC using the following process. First, we divided the PC axis into eight equally sized segments using the maximum and minimum values as boundaries. At each segmentation point, we identified candidate paintings with projected values within a ±0.015 range along the PC axis. Finally, we selected the painting with the minimum perpendicular distance to the PC axis among these candidates to minimize the fluctuation effect from other PCs.

### Estimation of Embedded Vectors’ Year Predictability Using XGBoost.

We evaluated the temporal expressibility of two vector representations by regression analysis using XGBoost, a scalable tree-boosting model (42). For each vector type (A- and C-vectors), we trained separate models using 70% of the original paintings as training data, with the respective latent vectors as input features to predict the painting years. The predictability of the models was measured using the remaining 30% of paintings. To verify the robustness of our results, we repeated this process 100 times with different random seeds for train-test splits, consistently obtaining similar performance across all iterations (see *SI Appendix*, Tables S6 and S7 for detailed statistics). These trained models were also used to estimate the temporal features of generated images, as shown in Fig. 4.

### Extraction of Human-Interpretable Latent Contexts from the Image.

To extract human-understandable contextual information from paintings, as we observed high expressibility of C-vectors, we employed the CLIP Interrogator (47), a widely used tool for prompt engineering for text-to-image models. CLIP Interrogator is designed to identify optimal generative prompts to resemble the input image. This tool integrates the CLIP model with the BLIP2 (48) image captioning model using a two-step process: first, an initial caption was generated using BLIP2, and then it was iteratively enhanced by incorporating predefined descriptive tokens (namely, flavors) to optimize the final prompt. There are five flavor categories: base flavors, artist names, media, movements, and negative. However, we excluded the artist names from the original flavor list because they directly indicate a specific artist’s style. If the prompt contained non-English letters, the name was normalized using Python’s internal unicodedata.normalize() function. All words composed of characters other than English letters and numbers were excluded from subsequent analysis. We measured the raw frequency using the number of unique words in each painting by counting duplicate words within a prompt (for the same artwork) as a single occurrence (for Fig. 3 and *SI Appendix*, Fig. S14 and Tables S1 and S2).

### Generative Experiment with SDM.

We investigated whether the observed temporal contextual patterns guided the evolutionary trajectory of Western paintings by designing a simple experiment using SDM’s image-to-image generation. We hypothesized that synthesizing paintings from a specific period within the primary contexts of the subsequent period may generate paintings in the style of the succeeding period. The experiment was performed using 500 paintings sampled from each century, generating new images from these source paintings using two distinct prompt conditions: 1) representative tokens from the consecutive century and 2) an empty prompt (‘’) serving as a null model without contextual guidance. We then predicted the estimated year of the generated images using the pretrained XGBoost regression model.

To identify the representative tokens for each century, we employed TF-IDF, calculated using scikit-learn’s TfidfVectorizer. Here, the TF-IDF score for text t in document d (where each document is a painting in this study) is as follows:

[2] $$\begin{array}{c} tf\text{-}idf(t,d)=tf(t,d)\times idf(t), \end{array}$$

where tf(t,d) represents the term frequency of text t in document d. The inverse document frequency idf(t) is defined as

[3] $$\begin{array}{c} idf\left(t\right)=\;\log\frac{1+n}{1+df(t)}+1, \end{array}$$

where n represents the total number of distinct paintings in the dataset, and df(t) denotes the number of paintings containing the term t. We first calculated the TF-IDF values of words in the generated prompt, where an artwork corresponds to a document. These values were then aggregated by computing the sum of the TF-IDF scores for each word across 100-y periods. We determined the word’s representative century based on the period in which it showed the highest TF-IDF value. We then selected the top 100 words using the TF-IDF score for each century. After sorting these words using their TF-IDF values, we manually filtered out words that may directly inject style information: 1) artist names, 2) names of art movements or style periods, and 3) numerical values (which might directly reference specific years). From the remaining words, we selected the top 77 words because CLIP’s text encoder has a maximum token length of 77 (41). Because the minimum number of tokens that can be generated from 77 words is exactly 77, selecting this number guaranteed the utilization of “at least” 77 tokens (see *SI Appendix*, Table S5 for the detailed word list). We then generated space-separated prompts from these tokens as “keyword_1_ keyword_2_ ⋯ keyword_77_” for Fig. 4. We also tested a comma-separated version of the prompt as “keyword_1_, keyword_2_, ⋯, keyword_77_,” with the results shown in *SI Appendix*, Fig. S16. Because commas are counted as separate tokens, the actual number of keywords used in the comma-separated version was reduced. However, commas may prevent words from being interpreted as connected phrases, providing clearer semantic boundaries between individual keywords. Note that any tokens appearing after this limit are automatically ignored by the model.

The strength parameter, defined between 0 for minimum perturbation and 1 for maximum perturbation in the SDM image-to-image model, determines the degree of transformation from the source image to the generated output. This parameter is used internally to control the noise addition process within the DDIM Solver (66), which serves as a noising–denoising process. Specifically, it first establishes the total number of steps required to reach the maximum noise level (i.e., DDIM steps) and then controls how many of these processes will be applied to the source image (i.e., the diffusion steps). Here, the diffusion step is determined as the product of DDIM steps and strength; naturally, this value is rounded when not an integer. Consequently, changes in strength do not affect the degree of perturbation if the difference is less than 1/(DDIM steps). Therefore, we adjusted the degree of transformation by directly changing the diffusion step rather than modifying the strength parameter with fixed DDIM steps (=50).

We also calculated the temporal axis $v_{1500s\to 1900s}$ as the difference between the average C-vectors of all paintings from the 1500s ($v_{1500s}$) and that from the 1900s ($v_{1900s}$), i.e., $v_{1500s\to 1900s}=v_{1900s}-v_{1500s}$. This vector represents the directional shift in contextual features across these periods.

## Supplementary Material

Appendix 01 (PDF)

## Data Availability

Code and processed data have been deposited in GitHub [https://github.com/aljinny/art-history (67)]. Previously published data were used for this work [https://deepart.hkust.edu.hk/ART500K/art500k.html (36)].
