# Supplementary material for: Context-aware multimodal AI navigates hidden pathways in five centuries of art evolution
Source: Proc Natl Acad Sci U S A. 2026 Jul 24;123(30):e2517969123. doi: 10.1073/pnas.2517969123 (PMC13416963; doi:10.1073/pnas.2517969123)
Supplement: Supplementary file 1 — Appendix 01 (PDF) [file pnas.2517969123.sapp.pdf]

Supporting Information for

Context-aware Multimodal AI Navigates Hidden Pathways in Five Centuries of Art Evolution

Jin Kim, Byunghwee Lee, Taekho You, and Jinhyuk Yun

Taekho You
E-mail: taekho.you@kaist.ac.kr
Jinhyuk Yun
E-mail: jinhyuk.yun@ssu.ac.kr

This PDF file includes:

Figs. S1 to S23
Tables S1 to S7
SI References

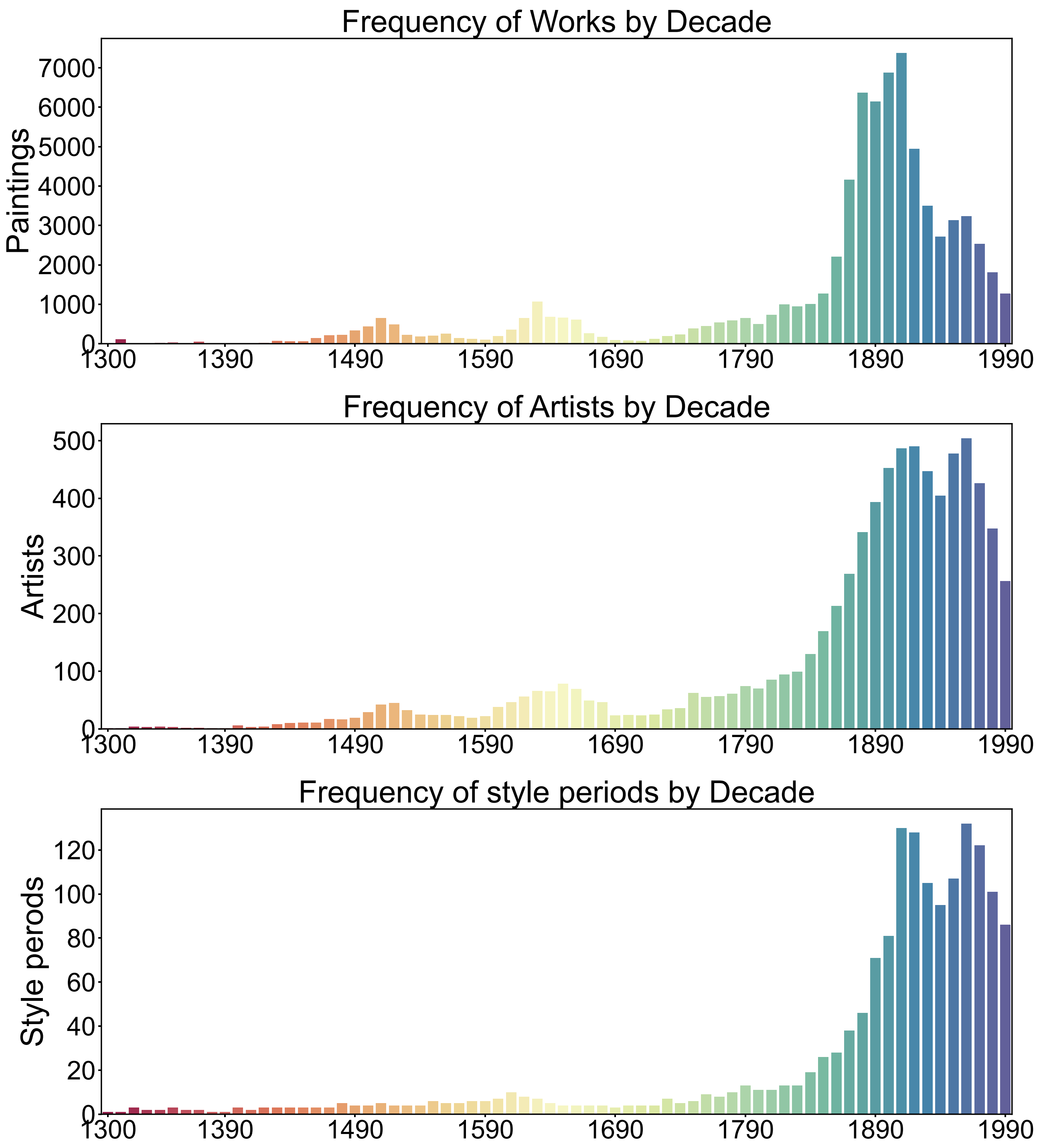

Fig. S1. Frequency of paintings, unique artists, and unique style periods from 1300 to 1990 by decade.

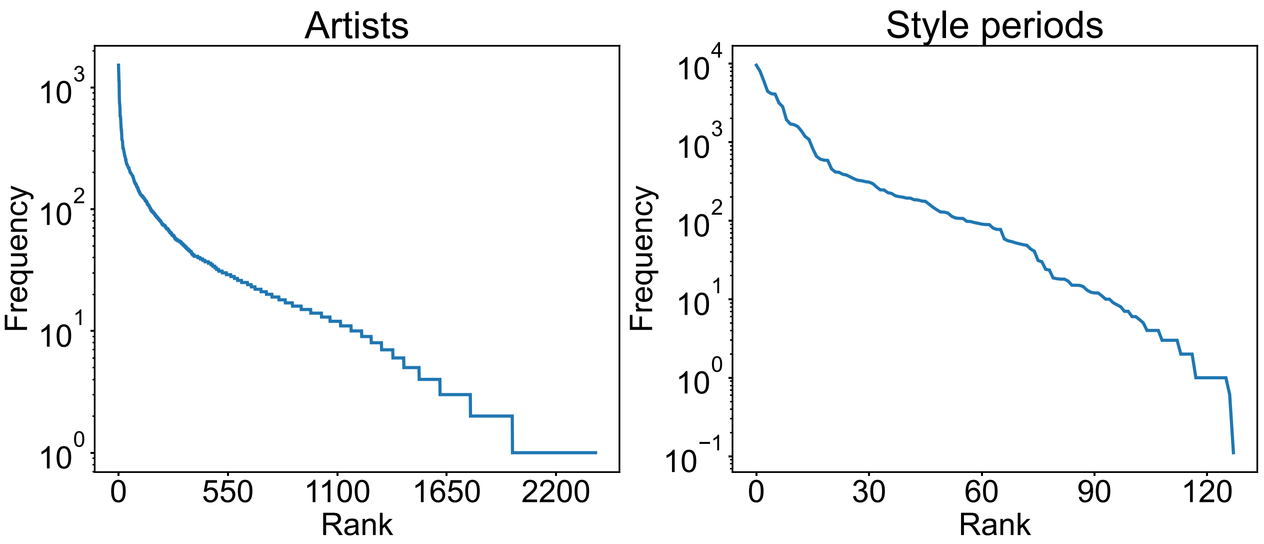

Fig. S2. Rank frequency of artists and style periods. The distribution is displayed in log-y scale.

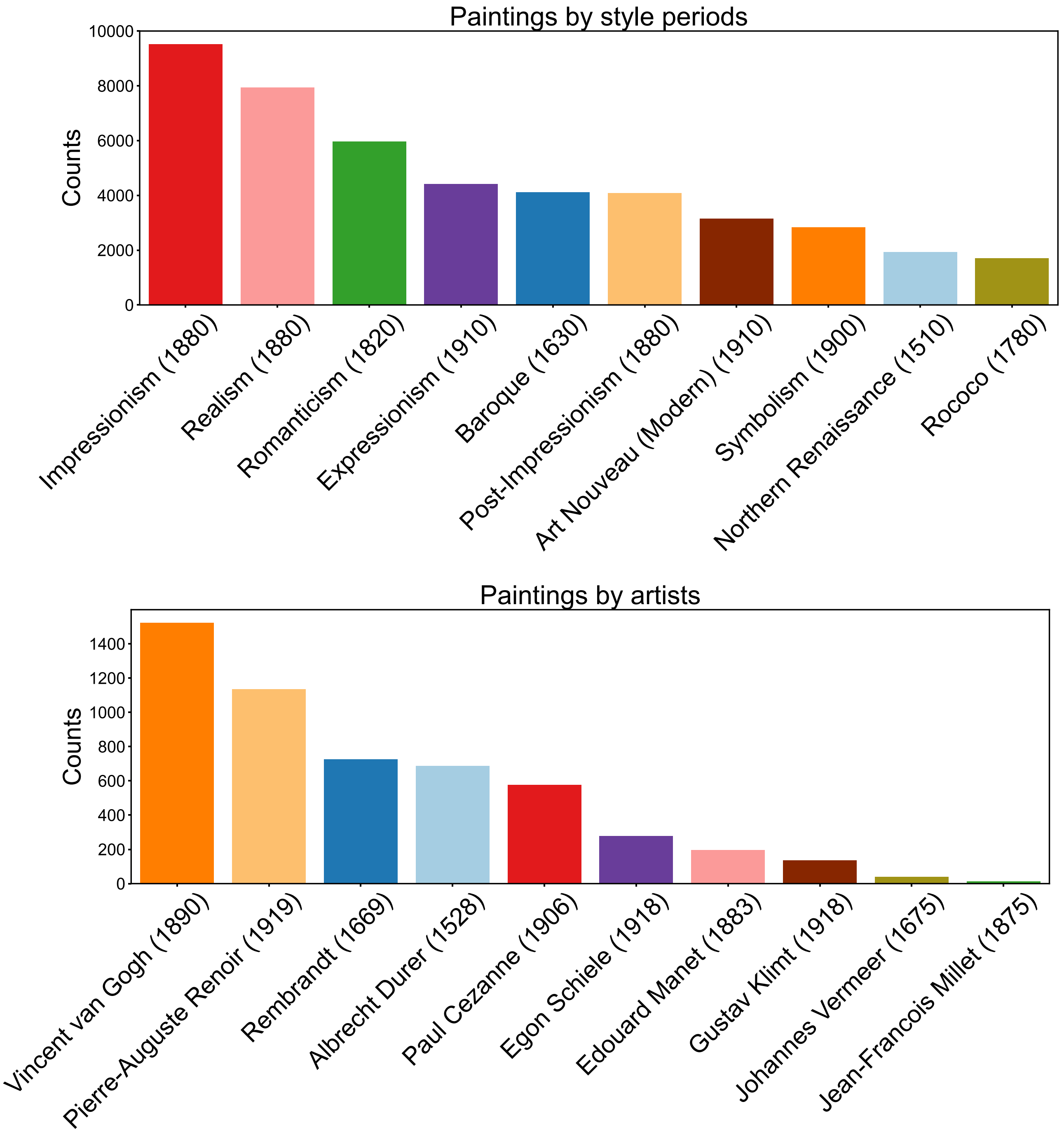

Fig. S3. Number of works by selected 10 style periods and artists. Style periods are chosen based on the number of paintings, while artists are chosen based on their recognition among the general public.

original

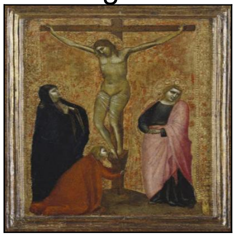

generation

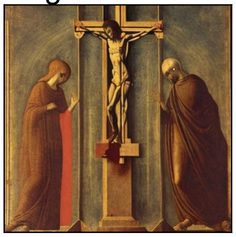

Allegretto Nuzi - Crucifixion With The Virgin Mary St John The Evangelist And St Mary Magdalene

Prompt

a painting of a crucif with three women and a man, by Lattanzio Gambara, nes, pedestal, 🔞 🤡, by Duccio, - i, high détails, momento mori, artststion, reyezuelo listado, northern renaissance, emil ferriss, album, springtime, scarlet, kami, trinity, gallows

original

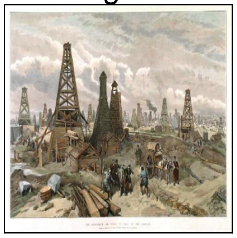

generation

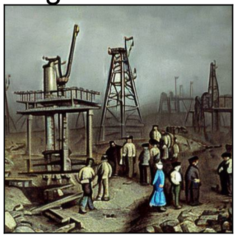

William Simpson - The Petroleum Oil Wells At Baku On The Caspian 1886

Prompt

there are many people walking around a large oil well, 1 9 century, ruined cities, chicago, pylons, 6 4 0, w 1024, the fashion of the time, lots of diamonds unearthed, chinese village, bizarrrrre, west slav features, oil slick

original

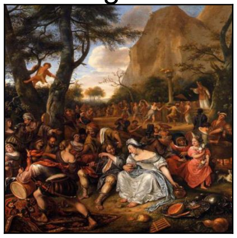

generation

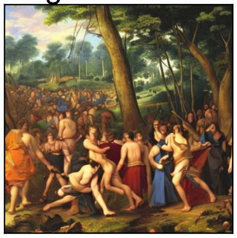

Jan Steen - Worship Of Golden Calf 1677

Prompt

a painting of a group of people in a wooded area, epic scene of zeus, revellers, dutch, background of invading army, cute woman, wikimedia commons, belgium, realistic scene, river of wine, impressive detail, contemporary art, ethnic, a fat, injured, harmony of

Fig. S4. Examples of regenerated artworks with prompts using the SDM, where the prompts were extracted from the original paintings using CLIP Interrogator (1).

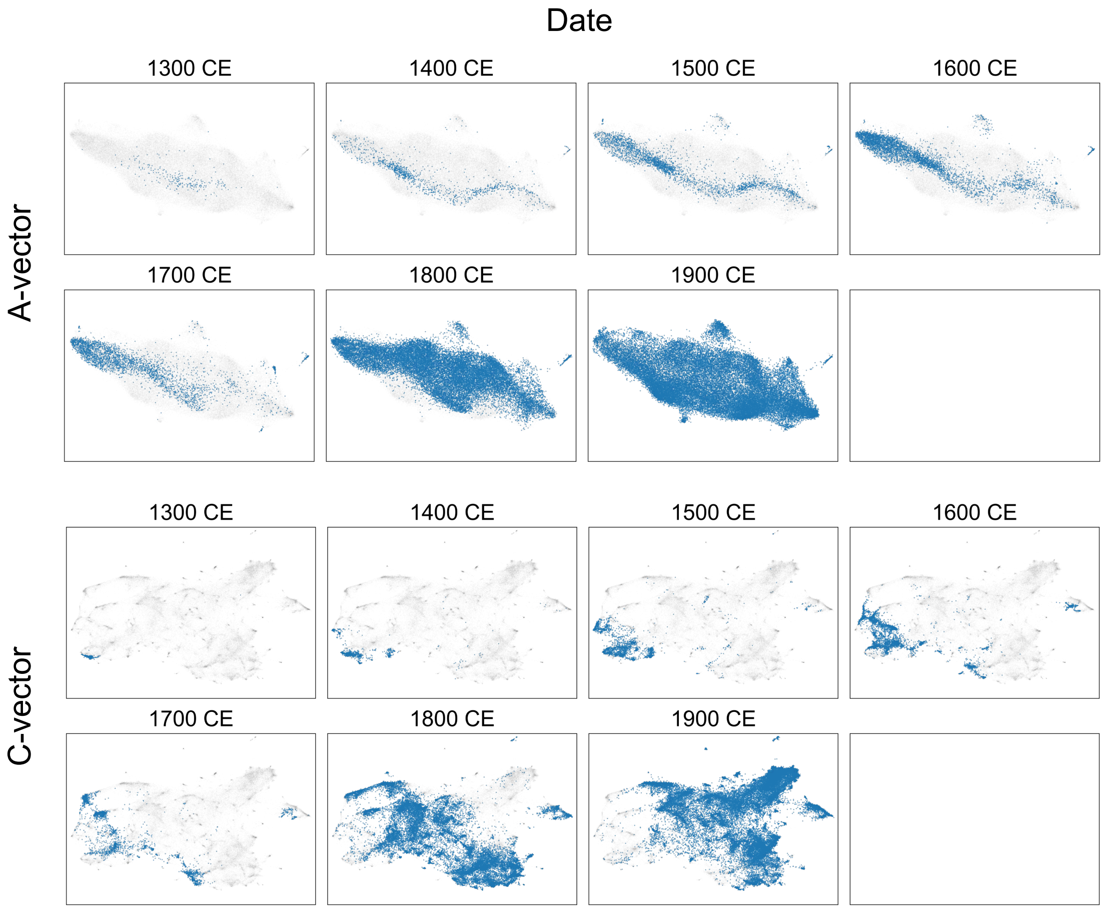

Fig. S5. Separated visualization of A- and C-vectors by year using UMAP.

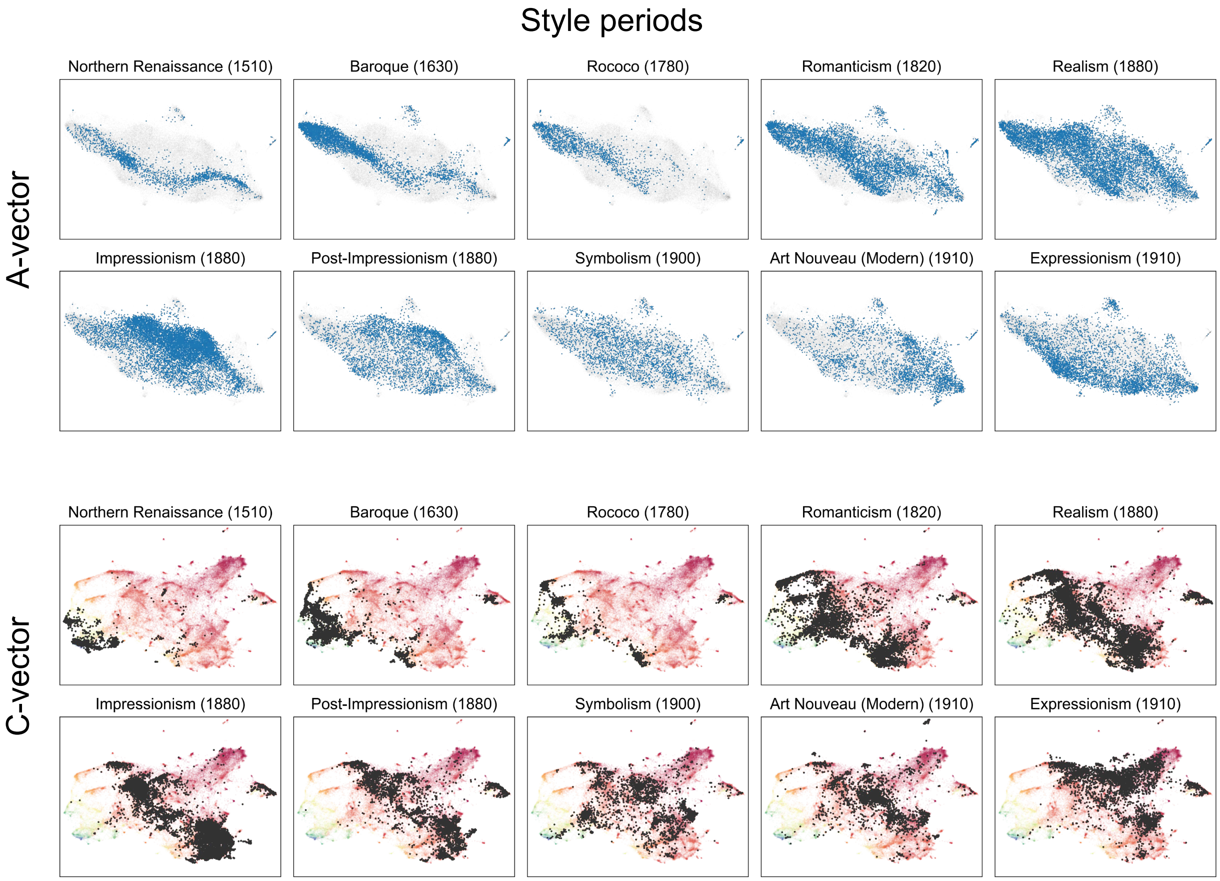

Fig. S6. Separated visualization of A- and C-vectors by style period using UMAP. To better visualize the relationship between style periods, which correspond to specific temporal periods, and the temporal progression in C-vectors, we overlaid paintings from specific styles onto the year heatmap from Fig. 1C.

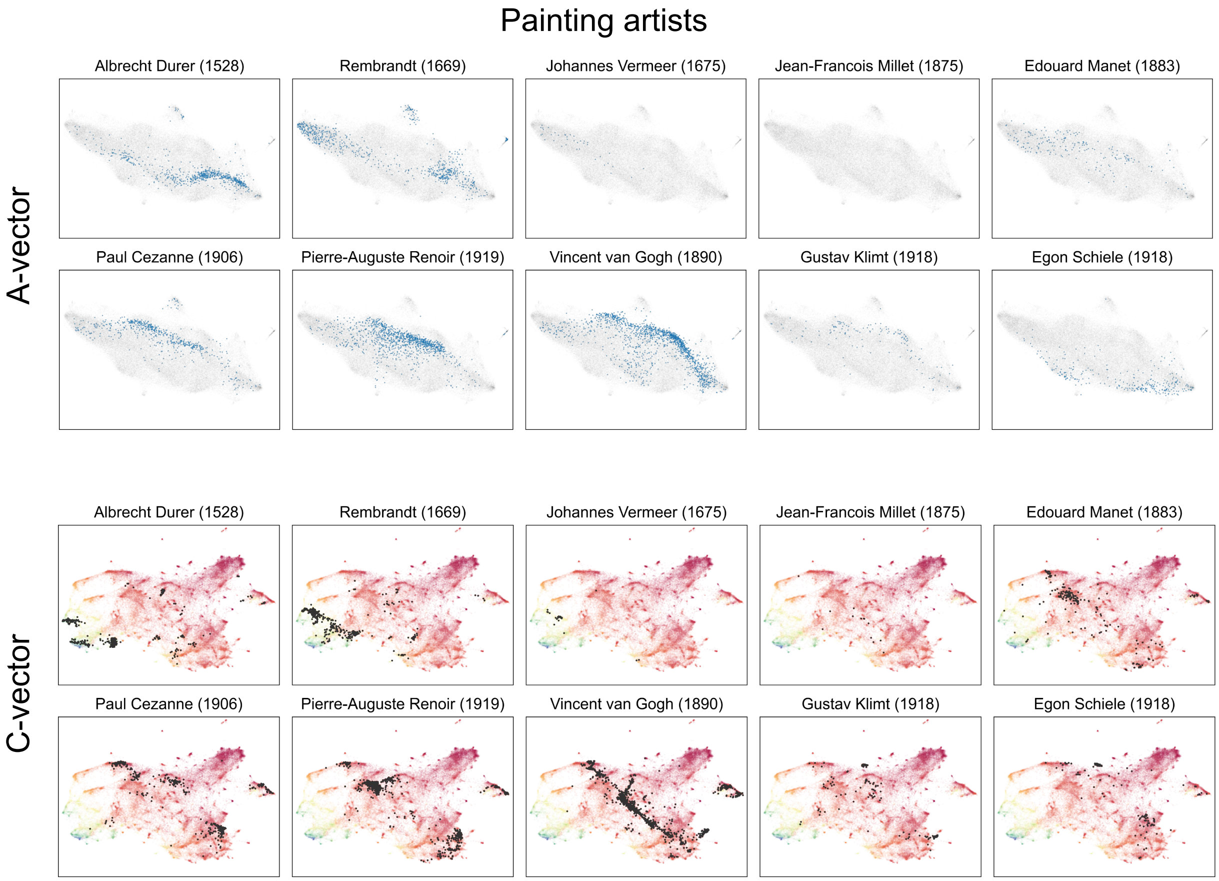

Fig. S7. Separated visualization of A- and C-vectors by artist using UMAP. To better visualize how artists' active periods relate to the temporal progression in C-vectors, we overlaid paintings from specific artists onto the year heatmap from Fig. 1C.

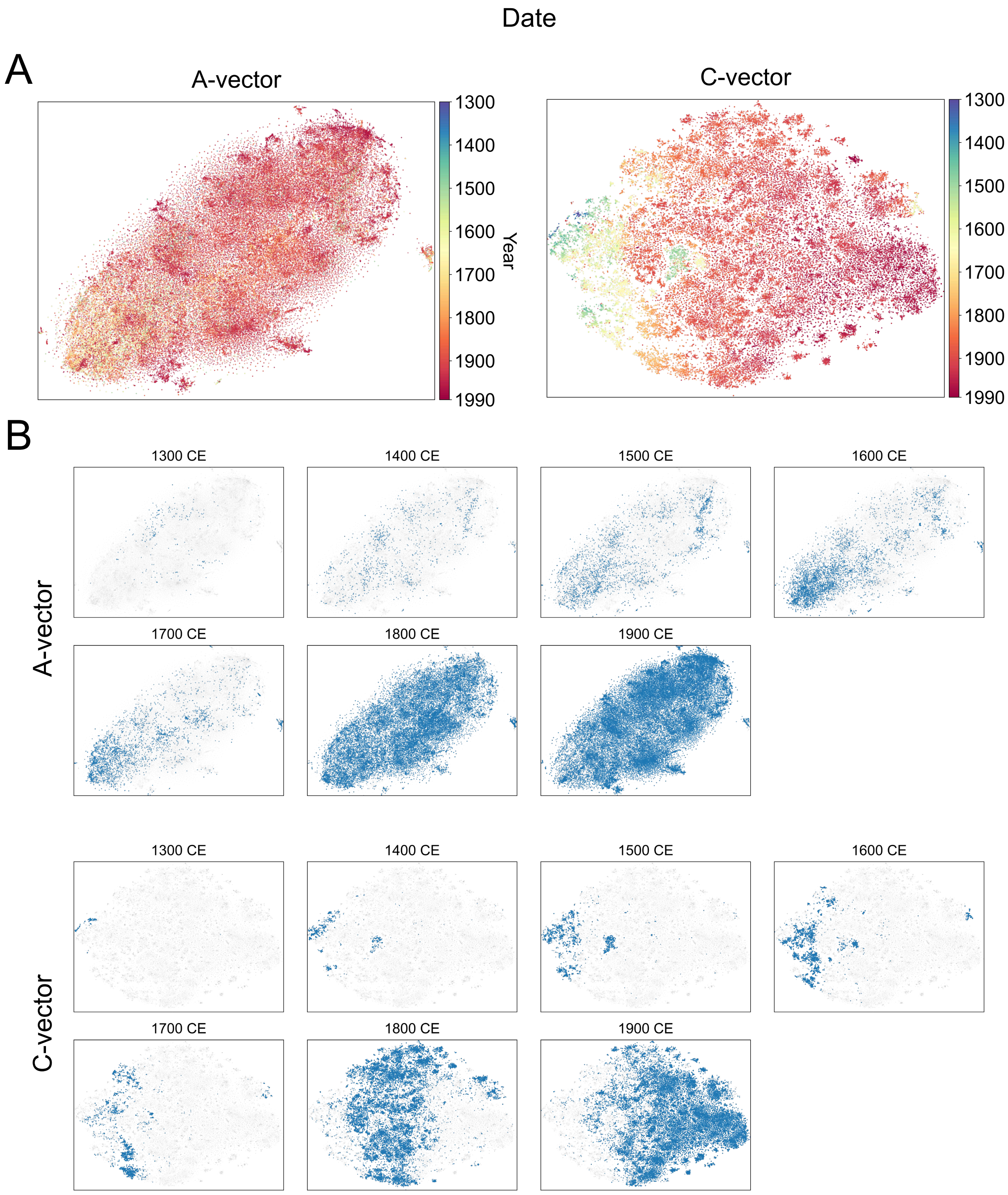

Fig. S8. **A**, Colored visualization of painting year distribution for A- and C-vectors using t-SNE. **B**, Separated visualization of A- and C-vectors by year using t-SNE.

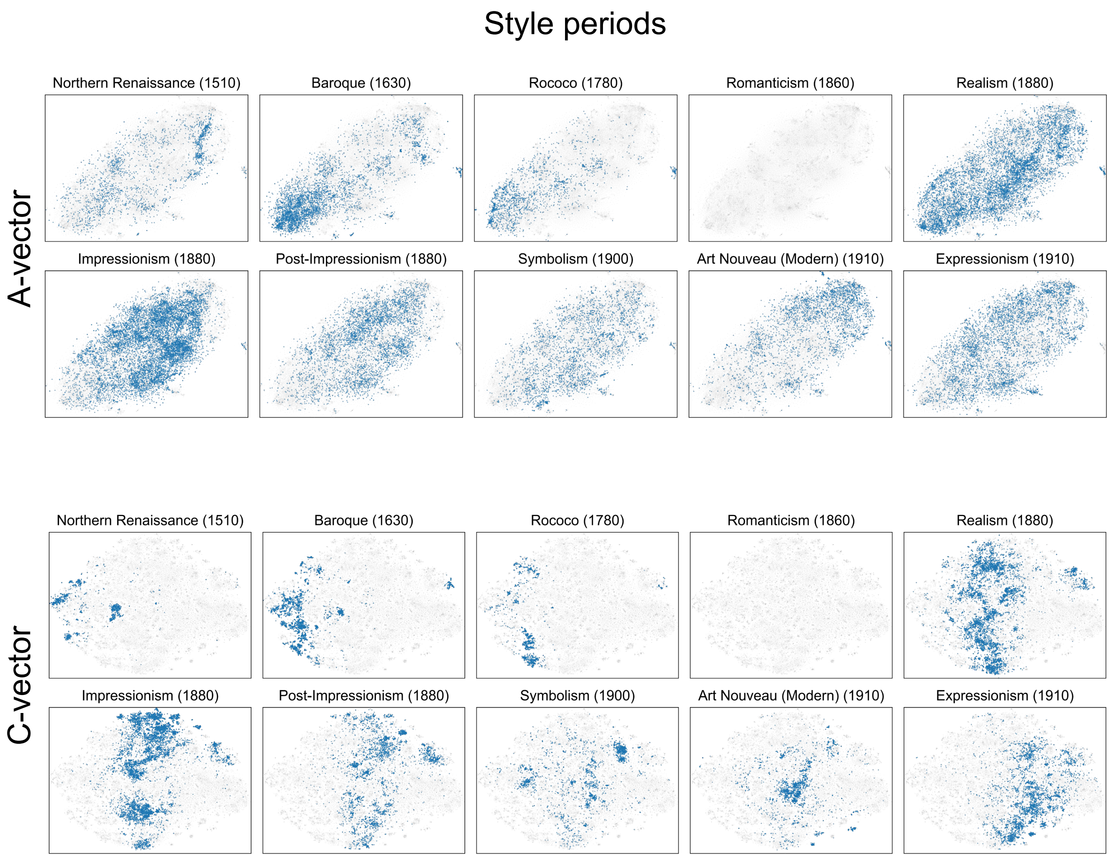

Fig. S9. Separated visualization of A- and C-vectors by style period using t-SNE.

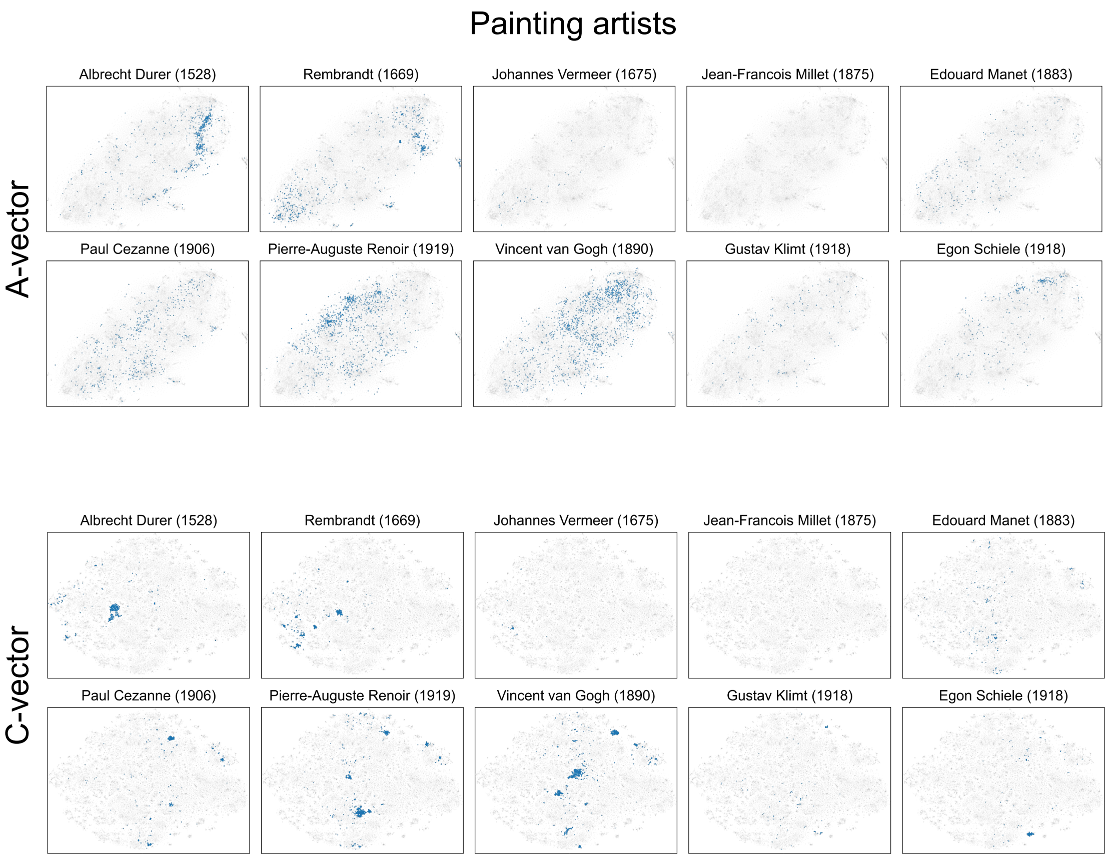

Fig. S10. Separated visualization of A- and C-vectors by artist using t-SNE.

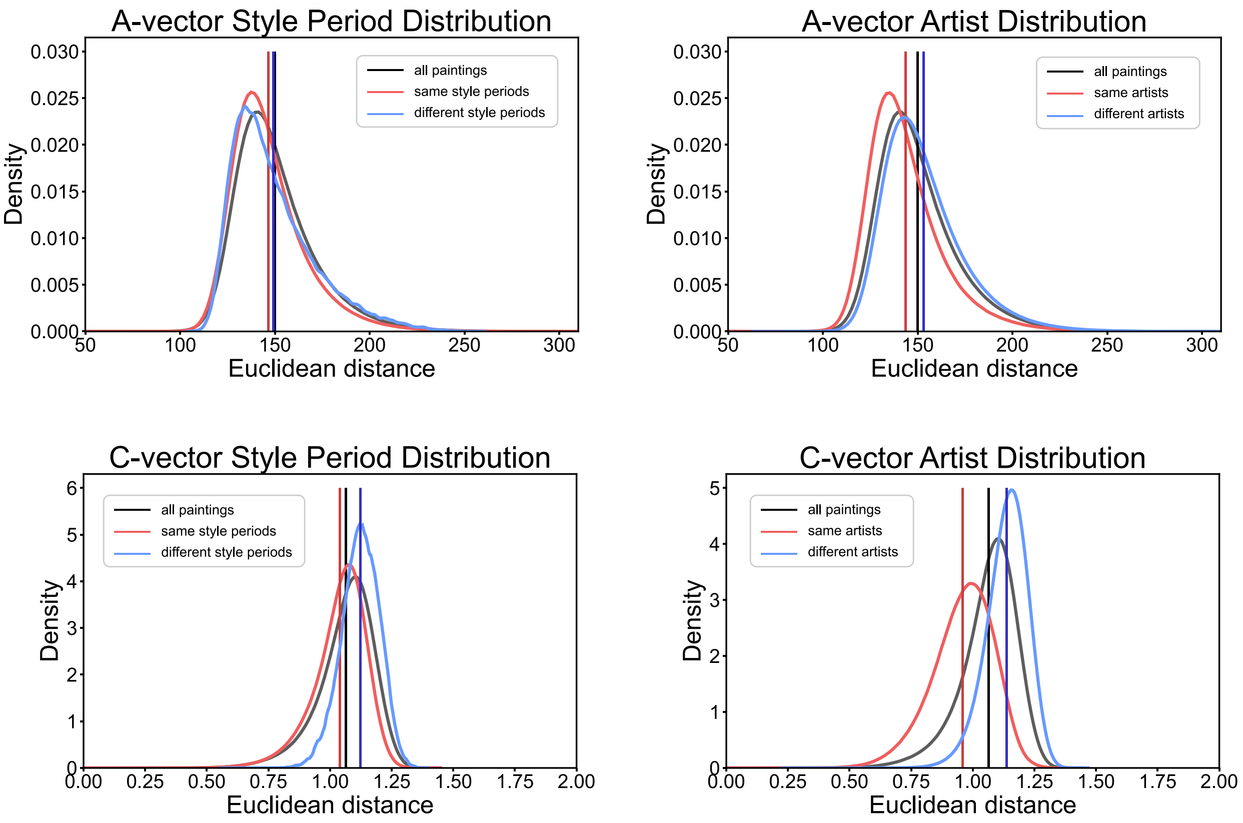

Fig. S11. Distance distributions of paintings' embedded vectors. C-vectors show a significantly larger separation between different authors (mean distance: 1.138 versus 0.959 for different and same authors, respectively, with 18.7% difference) than A-vectors (mean distance: 153.010 versus 143.753 for different and same authors, respectively, with 6.4% difference). This better discriminative power is also confirmed by the Kolmogorov-Smirnov (KS) test, where C-vectors show a substantially higher KS statistic between the same and different author distributions (0.614) compared to A-vectors (0.206). C-vectors also exhibit more consistent measurements across different styles (KS statistic: 0.330 versus 0.053 for A-vectors; mean distance: 1.122 versus 1.039 for C-vectors, whereas those for A-vectors are 149.150 versus 146.631). Note that the p-values from the KS tests were consistently 0.0 for all panels.

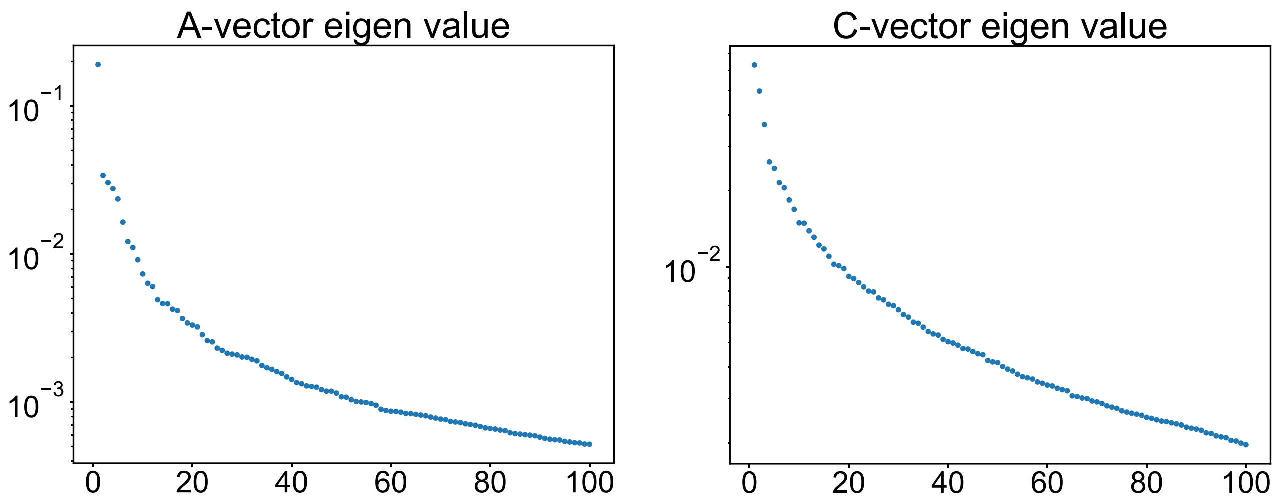

Fig. S12. Eigenvalue distribution of top 100 PCs in A- and C-vectors. The vertical axis is the eigenvalue for each PC, while the horizontal axis is its rank. Note that the vertical axes are displayed in the log scale.

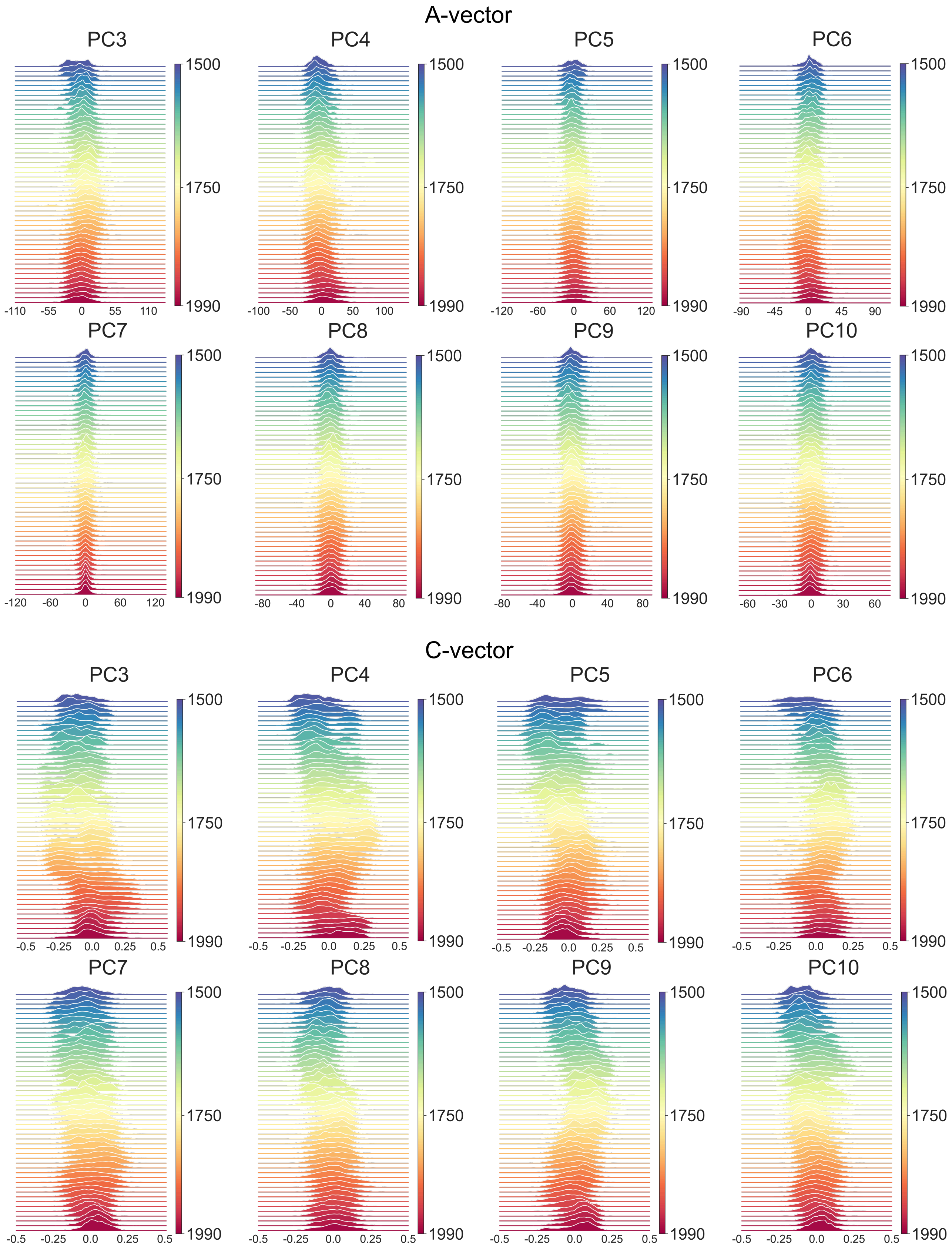

Fig. S13. Distribution of magnitudes on each of the top 10 PCs across painting years. For both A- and C-vectors, the horizontal axis is bounded by the minimum and maximum projected values on each PC axis.

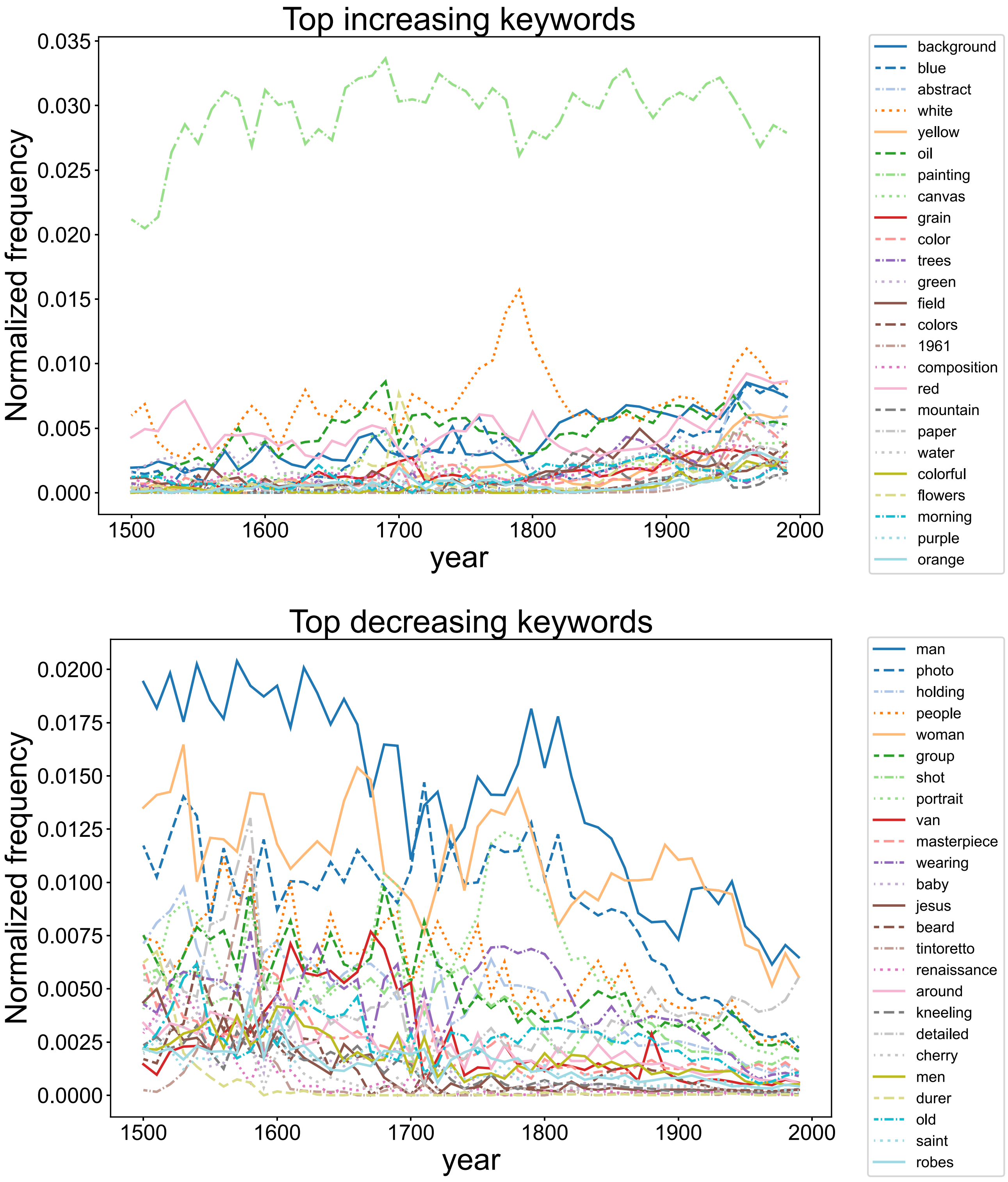

Fig. S14. Top increasing and decreasing keywords between 1500 and 2000. The degree of change is measured by the slope a of linear regression under the model $f(y) = ay + b$, where $f(y)$ is the L1-normalized frequency of the keyword at year y. Detailed slopes are listed in Tables S1 and S2.

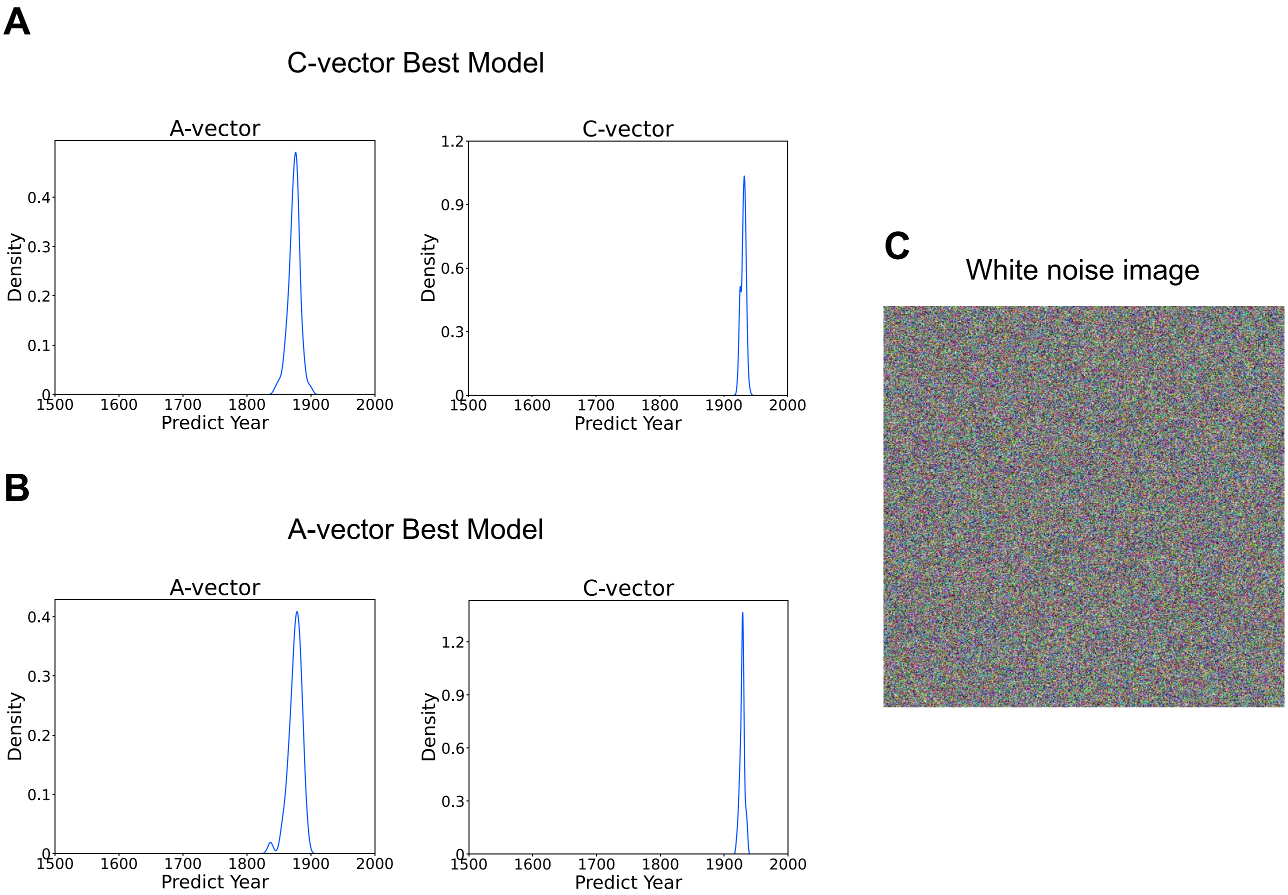

Fig. S15. Estimated year distribution of 100 randomly generated pure noise patterns using XGBoost models trained for Fig. 1. **A**, Predictions using the best C-vector model. **B**, Predictions using the best A-vector model. Noise images were randomly generated with the following Python code: `Image.fromarray((numpy.random.rand(512, 512, 3)*255).astype(numpy.uint8))` (see the sample image in **C**).

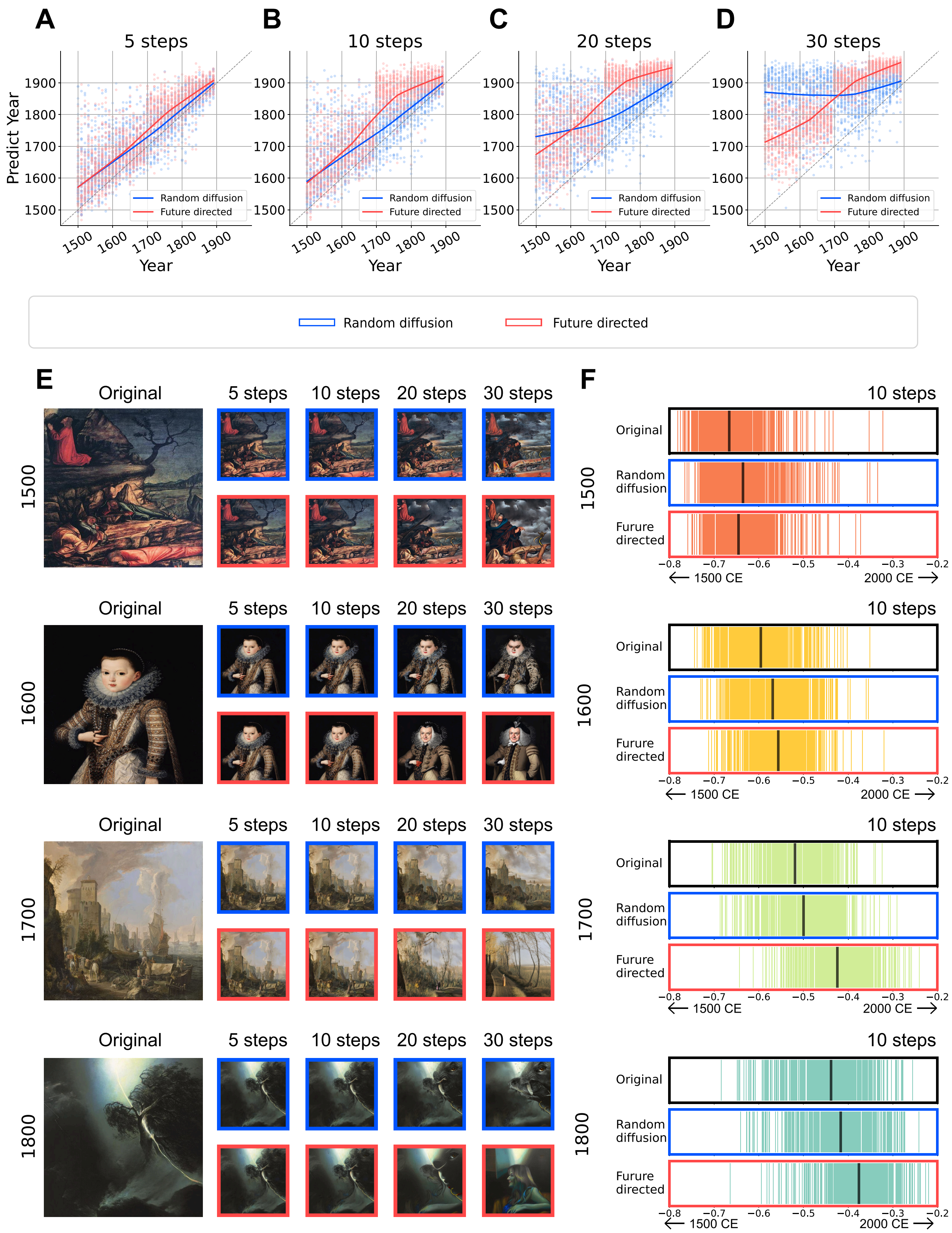

Fig. S16. Similar to Fig. 4, except that commas are used in the prompt generation process with 77 keywords representing the next century (e.g., $\texttt{keyword}_1$, $\texttt{keyword}_2$, $\cdots$, $\texttt{keyword}_{77}$). Because CLIP's tokenizer counts a comma as a separate token, the effective number of keywords in this prompt is reduced compared to prompts without commas, but this approach can avoid the possibility of individual words being used as compound phrases. Here, we also used the best-performing C-vector regression model to predict the years of generated paintings.

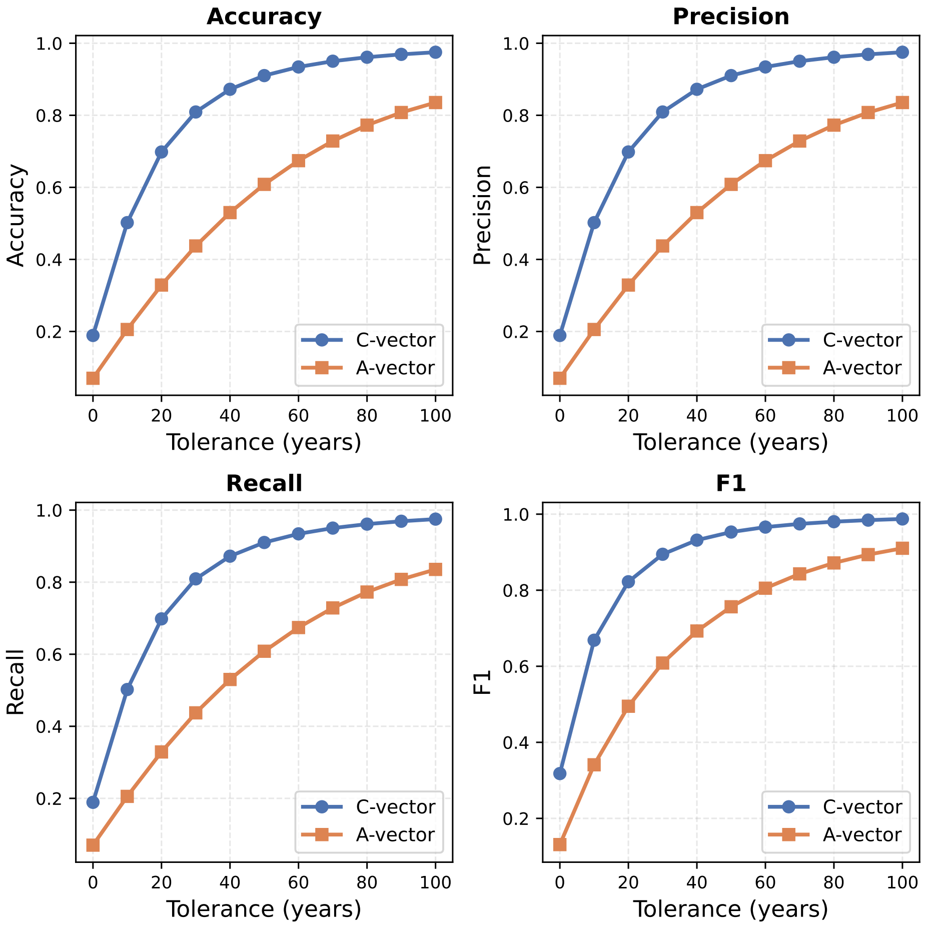

Fig. S17. Classification performance of A-vectors and C-vectors for predicting artwork creation periods across different error tolerances. Four classification metrics (Accuracy, Precision, Recall, and F1 score) are shown as functions of tolerance levels measured in years. Each metric is averaged across 100 XGBoost regression models (same models as Fig. 1) trained on A-vectors (orange) and C-vectors (blue) to predict artwork creation years, which were then binned into decades for classification evaluation. Tolerance 0 represents exact decade predictions, while tolerance n allows predictions within $\pm n$ years (e.g., tolerance 20 years means predictions within ± 2 decades are considered correct). Shaded regions indicate standard deviations across the 100 models, though these are small and barely visible, demonstrating the robustness of our regression models. C-vectors consistently outperform A-vectors across all metrics and tolerance levels, demonstrating superior capability in capturing temporal information.

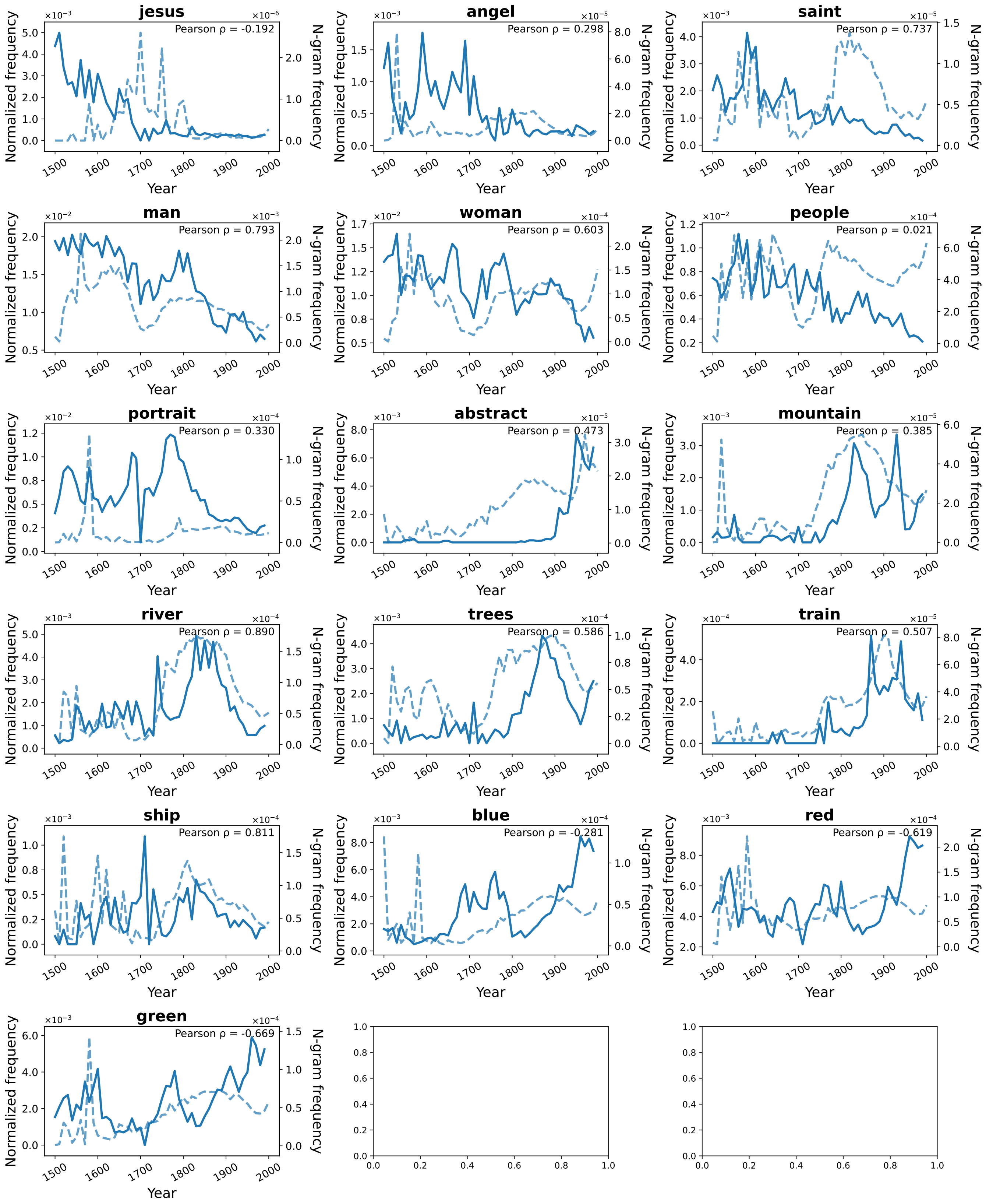

Fig. S18. Relationship between generated prompts and Google Books N-gram frequency. Temporal evolution of selected words comparing AI-generated prompts from paintings (solid lines; same as Fig. 3) and Google Books N-gram normalized frequencies (dashed lines). Google N-gram data were averaged by decade from yearly values. Pearson correlation coefficients between the two time series are displayed in the upper right of each panel. For visual comparison, both y-axes were set with 10% margins beyond their respective maximum and minimum values. Google Books N-gram data were collected using the Google Books API (https://books.google.com/ngrams/json) on October 13, 2025.

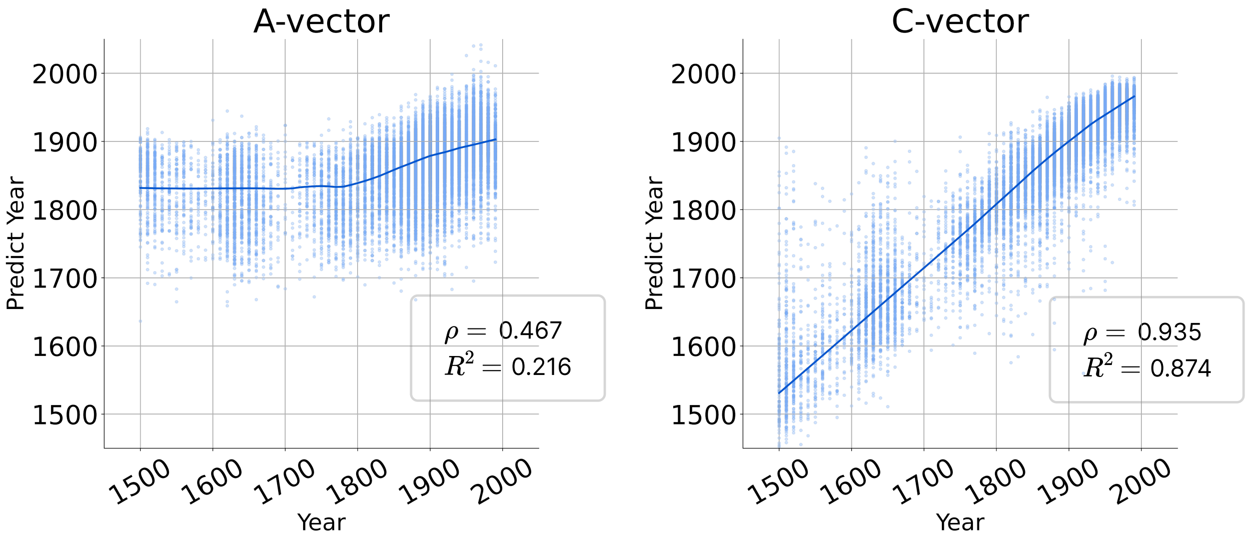

Fig. S19. Regression performance of the best A-vector model among 100 train-test splits for predicting artwork creation years. Experimental setup follows Fig. 1H,I, which shows the best C-vector model.

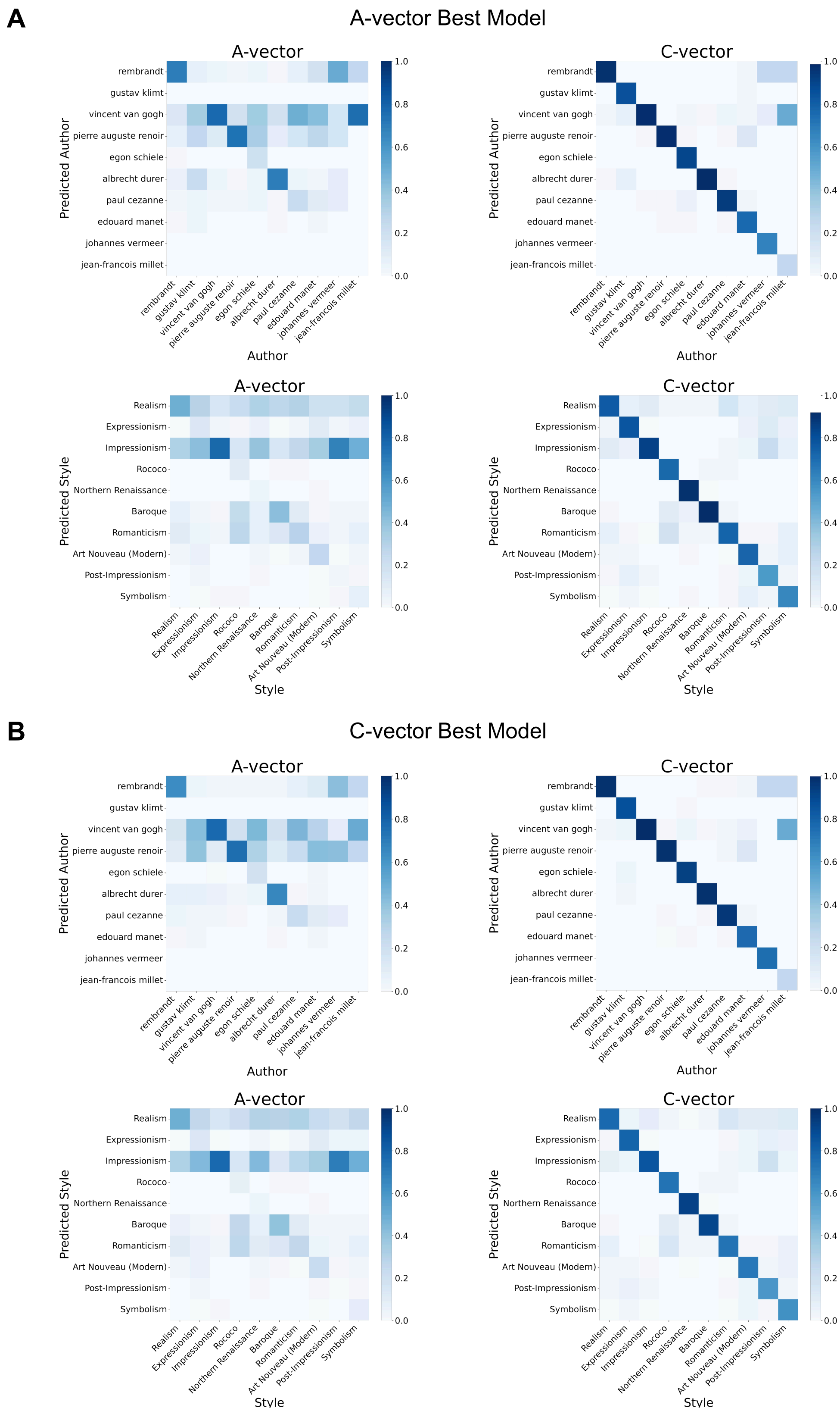

Fig. S20. Confusion matrices comparing classification performance between A-vectors and C-vectors across 100 train-test splits. **A**, Results from the best-performing A-vector model for predicting 10 artists (top) and 10 art movements (bottom), showing moderate accuracy with substantial off-diagonal misclassifications. **B**, Results from the best-performing C-vector model, demonstrating better classification with stronger diagonal patterns and reduced confusion across both artists and art movements. Note that performance is consistent across all 100 train-test split configurations (see SI Appendix, Tables S7).

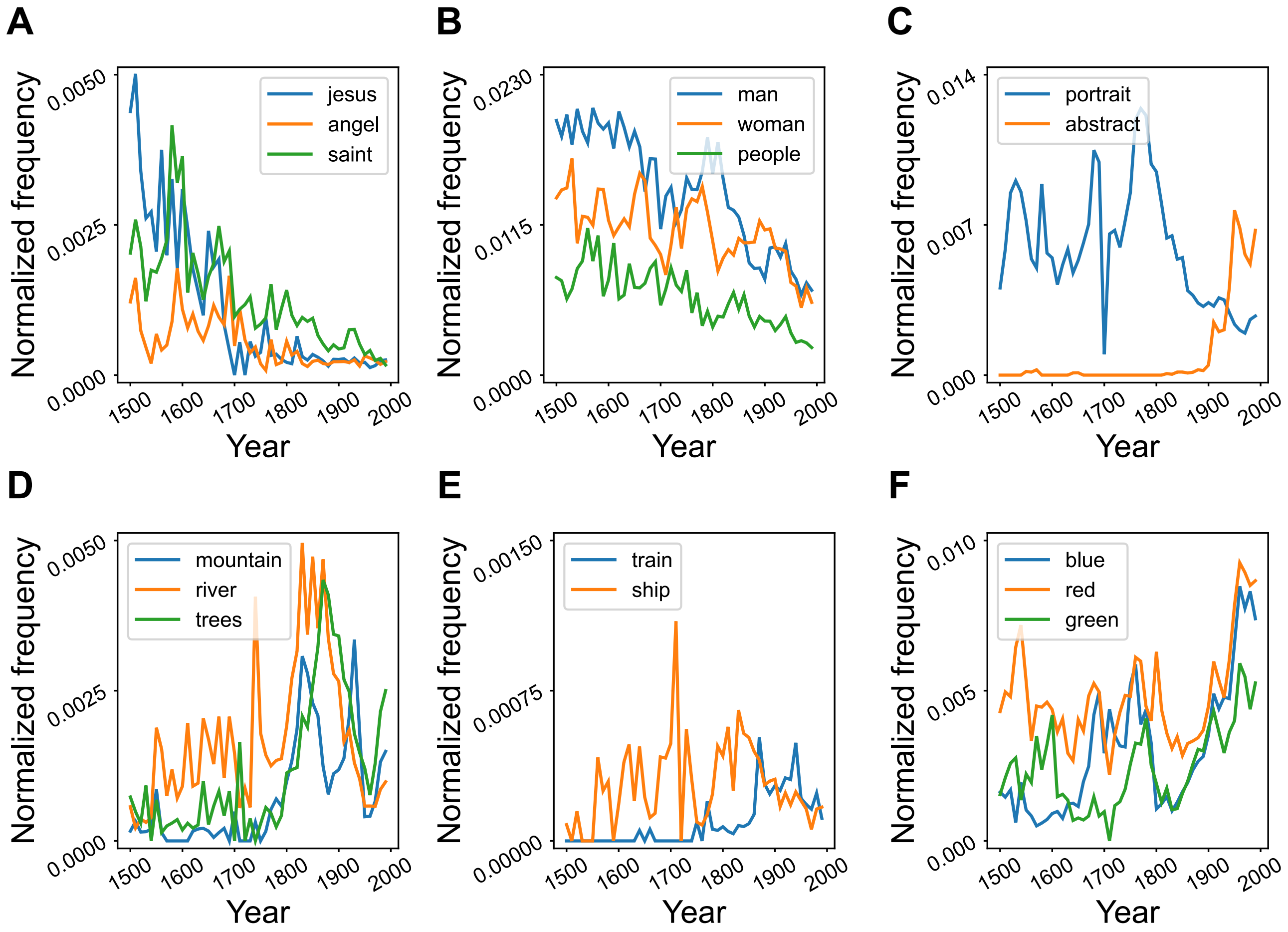

Fig. S21. Temporal patterns of generative keywords in Fig. 3, after removing style-specific keywords. To address potential confounding effects from style-related terms that vary across periods, we tested the robustness of our results by excluding 412 style-specific words that appeared in the style column but not in the genre column of Art500k dataset. Of these 412 words, only 32 actually appeared in our dataset, accounting for $7\,340$ occurrences out of $2\,002\,204$ total keyword frequencies ($\sim$0.37%). This minimal impact confirms that our observed temporal patterns are robust against style-related noise.

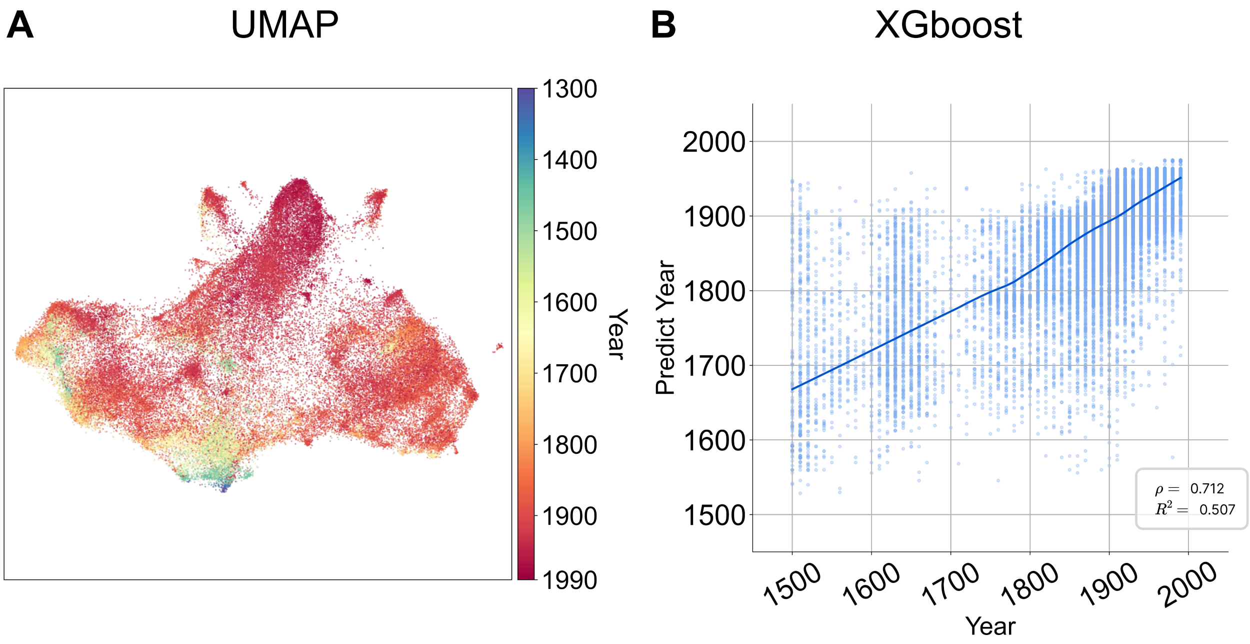

Fig. S22. Temporal structure of SynthCLIP vectors. Results are obtained using a ViT-B/16 model trained on SynthCLIP-30M (2), which was trained exclusively on synthetically generated image-text pairs. A, A two-dimensional (2D) projection of 72,447 Western paintings was obtained using UMAP with SynthCLIP vectors, where each dot represents a painting and dot colors indicate painting years. B, The results of the XGBoost regression model predicting painting year from SynthCLIP vectors, using the same train/test split as the best-performing C-vector model shown in Fig. 1. The model obtains $R^2 = 0.507$ and Pearson $\rho = 0.712$, substantially higher than the A-vector baseline ($R^2 = 0.202$, Pearson $\rho = 0.450$).

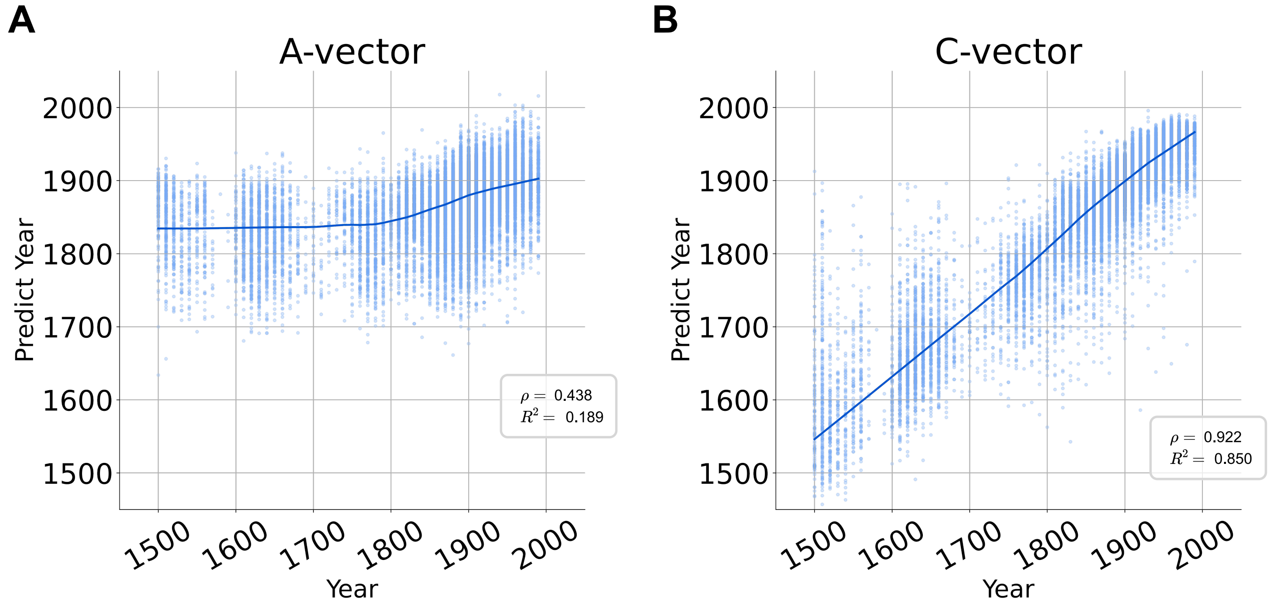

Fig. S23. XGBoost year regression results based on artist-based train/test split. To address potential artist confounding effects in the original random split, e.g., a model learning what a "Van Gogh" painting looks like and predicting the corresponding time period rather than learning temporal patterns per se, we conducted a new experiment using an artist-based split, where each artist is assigned to a 100-year bin based on the median year of their paintings. Within each bin, the artist list is shuffled using a fixed random seed (3337), and partitioned to most closely approximate a 70/30 split by painting count, resulting in a training set of 1,618 artists and 50,556 paintings (69.8%) and a test set of 735 artists and 21,891 paintings (30.2%). This design ensures that no artist appears in both training and test sets, while preserving temporal balance across centuries. A, XGBoost regression results for A-vectors, achieving $R^2 = 0.189$ and Pearson $\rho = 0.438$. B, XGBoost regression results for C-vectors, achieving $R^2 = 0.850$ and Pearson $\rho = 0.922$. Both results are remarkably consistent with the original random split findings (A-vector mean: $R^2 = 0.202$, $\rho = 0.450$; C-vector mean: $R^2 = 0.869$, $\rho = 0.932$), demonstrating that the substantial performance gap between A- and C-vectors is not an artifact of artist confounding.

Table S1. Top increasing keywords between 1500 and 2000, ordered by slope. The degree of increment is measured by the slope a of linear regression under the model $f(y) = ay + b$, where $f(y)$ is the L1-normalized frequency of the keyword at year y.

Rank	Word	Slope	Rank	Word	Slope
1	background	1.23×10^-5	51	soft	1.96×10^-6
2	blue	1.02×10^-5	52	distance	1.95×10^-6
3	abstract	8.82×10^-6	53	summer	1.91×10^-6
4	white	8.75×10^-6	54	turner	1.87×10^-6
5	yellow	8.66×10^-6	55	colours	1.87×10^-6
6	oil	7.85×10^-6	56	person	1.86×10^-6
7	painting	7.24×10^-6	57	shapes	1.85×10^-6
8	canvas	6.12×10^-6	58	colour	1.84×10^-6
9	grain	5.26×10^-6	59	russian	1.82×10^-6
10	color	5.12×10^-6	60	impasto	1.82×10^-6
11	trees	5.12×10^-6	61	deep	1.79×10^-6
12	green	4.79×10^-6	62	evening	1.78×10^-6
13	field	4.71×10^-6	63	courtesy	1.77×10^-6
14	colors	4.35×10^-6	64	pattern	1.72×10^-6
15	1961	4.18×10^-6	65	road	1.71×10^-6
16	composition	4.11×10^-6	66	frankenthaler	1.70×10^-6
17	red	4.05×10^-6	67	helene	1.70×10^-6
18	mountain	3.80×10^-6	68	monet	1.69×10^-6
19	paper	3.44×10^-6	69	forest	1.67×10^-6
20	water	3.19×10^-6	70	pale	1.67×10^-6
21	colorful	3.15×10^-6	71	unknown	1.61×10^-6
22	flowers	3.15×10^-6	72	todorovitch	1.58×10^-6
23	morning	3.09×10^-6	73	edward	1.57×10^-6
24	purple	3.07×10^-6	74	pastel	1.56×10^-6
25	orange	2.99×10^-6	75	kupka	1.55×10^-6
26	scheme	2.96×10^-6	76	plan	1.55×10^-6
27	paint	2.96×10^-6	77	mist	1.53×10^-6
28	river	2.84×10^-6	78	sketch	1.52×10^-6
29	walking	2.79×10^-6	79	bright	1.49×10^-6
30	house	2.79×10^-6	80	tree	1.47×10^-6
31	sky	2.78×10^-6	81	frank	1.46×10^-6
32	art	2.59×10^-6	82	jean	1.46×10^-6
33	beach	2.54×10^-6	83	park	1.43×10^-6
34	landscape	2.53×10^-6	84	print	1.42×10^-6
35	street	2.46×10^-6	85	snow	1.42×10^-6
36	vase	2.45×10^-6	86	design	1.42×10^-6
37	boat	2.42×10^-6	87	watercolor	1.42×10^-6
38	foreground	2.42×10^-6	88	horizontal	1.35×10^-6
39	square	2.42×10^-6	89	hill	1.35×10^-6
40	stained	2.40×10^-6	90	william	1.34×10^-6
41	ffffound	2.39×10^-6	91	1921	1.34×10^-6
42	pink	2.35×10^-6	92	day	1.33×10^-6
43	violet	2.26×10^-6	93	expressionism	1.33×10^-6
44	lines	2.22×10^-6	94	village	1.32×10^-6
45	chair	2.21×10^-6	95	tie	1.32×10^-6
46	john	2.14×10^-6	96	night	1.31×10^-6
47	wall	2.11×10^-6	97	bridge	1.31×10^-6
48	charles	2.07×10^-6	98	elevation	1.31×10^-6
49	city	2.03×10^-6	99	horizon	1.31×10^-6
50	cut	1.99×10^-6	100	garden	1.27×10^-6

Table S2. Top decreasing keywords between 1500 and 2000, ordered by the slope. The degree of decrement is measured by the slope a of linear regression under the model $f(y) = ay + b$, where $f(y)$ is the L1-normalized frequency of the keyword at year y.

Rank	Word	Slope	Rank	Word	Slope
1	man	-2.72×10^{-5}	51	fibbonacci	-2.64×10^{-6}
2	photo	-1.66×10^{-5}	52	young	-2.61×10^{-6}
3	holding	-1.30×10^{-5}	53	male	-2.53×10^{-6}
4	people	-1.18×10^{-5}	54	child	-2.52×10^{-6}
5	woman	-1.18×10^{-5}	55	hand	-2.50×10^{-6}
6	group	-1.09×10^{-5}	56	book	-2.43×10^{-6}
7	shot	-8.68×10^{-6}	57	album	-2.41×10^{-6}
8	portrait	-7.81×10^{-6}	58	massys	-2.38×10^{-6}
9	van	-7.77×10^{-6}	59	drawing	-2.36×10^{-6}
10	masterpiece	-7.45×10^{-6}	60	female	-2.35×10^{-6}
11	wearing	-7.33×10^{-6}	61	italian	-2.30×10^{-6}
12	baby	-7.31×10^{-6}	62	various	-2.28×10^{-6}
13	jesus	-7.19×10^{-6}	63	sienna	-2.26×10^{-6}
14	beard	-6.49×10^{-6}	64	madonna	-2.25×10^{-6}
15	tintoretto	-6.45×10^{-6}	65	intricate	-2.22×10^{-6}
16	renaissance	-6.21×10^{-6}	66	screen	-2.21×10^{-6}
17	around	-5.82×10^{-6}	67	rembrandt	-2.20×10^{-6}
18	kneeling	-5.74×10^{-6}	68	artists	-2.16×10^{-6}
19	detailed	-5.47×10^{-6}	69	taken	-2.11×10^{-6}
20	cherry	-5.35×10^{-6}	70	head	-2.07×10^{-6}
21	men	-5.16×10^{-6}	71	chest	-2.05×10^{-6}
22	durer	-5.11×10^{-6}	72	picture	-2.04×10^{-6}
23	old	-4.74×10^{-6}	73	short	-2.03×10^{-6}
24	saint	-4.72×10^{-6}	74	information	-2.02×10^{-6}
25	robes	-4.52×10^{-6}	75	640	-1.98×10^{-6}
26	high	-4.18×10^{-6}	76	cropped	-1.98×10^{-6}
27	dark	-4.02×10^{-6}	77	caravaggio	-1.94×10^{-6}
28	cross	-3.93×10^{-6}	78	michelangelo	-1.92×10^{-6}
29	image	-3.93×10^{-6}	79	angel	-1.89×10^{-6}
30	angels	-3.86×10^{-6}	80	bosch	-1.89×10^{-6}
31	paolo	-3.78×10^{-6}	81	hat	-1.89×10^{-6}
32	santa	-3.72×10^{-6}	82	black	-1.86×10^{-6}
33	sword	-3.69×10^{-6}	83	hd	-1.85×10^{-6}
34	veronese	-3.54×10^{-6}	84	listing	-1.84×10^{-6}
35	panel	-3.52×10^{-6}	85	crown	-1.82×10^{-6}
36	robe	-3.37×10^{-6}	86	ascending	-1.81×10^{-6}
37	titian	-3.27×10^{-6}	87	venetian	-1.79×10^{-6}
38	spring	-3.27×10^{-6}	88	palm	-1.78×10^{-6}
39	baroque	-3.22×10^{-6}	89	mantegna	-1.74×10^{-6}
40	silver	-3.12×10^{-6}	90	goddess	-1.74×10^{-6}
41	god	-3.07×10^{-6}	91	family	-1.73×10^{-6}
42	armor	-3.07×10^{-6}	92	beautiful	-1.72×10^{-6}
43	full	-3.03×10^{-6}	93	gondola	-1.71×10^{-6}
44	rubens	-2.96×10^{-6}	94	body	-1.69×10^{-6}
45	holy	-2.94×10^{-6}	95	angle	-1.67×10^{-6}
46	dutch	-2.88×10^{-6}	96	zurbaran	-1.67×10^{-6}
47	hands	-2.88×10^{-6}	97	trinity	-1.63×10^{-6}
48	arbor	-2.87×10^{-6}	98	pope	-1.60×10^{-6}
49	catalog	-2.73×10^{-6}	99	quality	-1.60×10^{-6}
50	years	-2.68×10^{-6}	100	dog	-1.59×10^{-6}

Table S3. Filtering criteria for removed keywords.

Label	Value
Style	Abbasid Period, Byzantine, Dictionaries with no category, Documentary photography, Ero guro, Folk art, Gongbi, Gothic, Ilkhanid, Indian, Ink and wash painting, Islamic Art, Japanese, Japonism, Joseon Dynasty, Kano school style, Korean Art, Mughal, Nanga (Bunjinga), Nas-Taliq, Native Art, New Ink Painting, Nihonga, Ottoman Period, Post-classic (c.900-1580), Pre-Columbian Art, Safavid, Safavid Period, Shin-hanga, Sosaku hanga, Street Photography, Sumi-e (Suiboku-ga), Kano school style, Timurid Period, Ukiyo-e, Yamato-e, Yoruba, Zen
Field	architecture, calligraphy, ceiling, chinese, digital, fresco, furniture, glass, installation, interior, japanese, masonite, oriental, photo, porcelain, sculpture, silk, ukiyo, wall, woodcut
Genre	advertisement, bijinga, graffiti, installation, performance, photo, sculpture, tapestry
Nationality	Armenian, Azerbaijani, Cameroonian, Chinese, Egyptian, Emirati, Ethiopian, Filipino, Georgian, Indian, Indonesian, Iranian, Iraqi, Japanese, Lebanese, Libyan, Nigerian, Qatari, South Korean, Sudanese, Syrian, Vietnamese

Table S4. Filtering criteria for selected keywords.

Label	Value
Field	canvas, charcoal, drawing, etching, gouache, ink, lithograph, oil, painting, paper, pastel, printmaking, sketch, tempera, watercolor

Table S5. Future-directed keywords for the diffusion experiments. After an initial selection process of the top 100 words by TF-IDF (see Materials and Methods), we manually removed 1) numbers and year-like formats (which may directly indicate a specific year), 2) artistic styles, and 3) known artists' names (red italic words). Blue boldface words represent the top-77 ranked words after manual filtering. Note that 77 tokens is the input limit of CLIP, and we used 77 words to ensure the maximum number of tokens for each century. Any prompt tokens appearing after the 77th token are ignored automatically by the model.

Year	1600s		1700s	
RANK	Words	TF-IDF	Words	TF-IDF
1	dutch	72.57	wig	25.56
2	gerit	44.03	powdered	19.13
3	angels	32.11	berne	16.33
4	fibbonacci	28.49	georgian	14.06
5	*1647*	23.71	garbage	13.41
6	*1666*	22.3	*1759*	13.05
7	chiaroscuro	21.7	cgsocietywlop	11.97
8	masters	19.57	doff	11.57
9	ascending	17.66	*1786560639*	10.76
10	toga	17.05	colonial	9.81
11	ope	16.65	palladian	9.31
12	*lieven*	13.29	f1	7.79
13	nylon	12.94	ancestral	7.5
14	zeus	12.58	euler	7.5
15	pauldrons	12.39	aerith	7.36
16	goliath	12.12	stathmore	7.13
17	klaarbergen	11.52	moravian	5.94
18	samma	11.52	britannia	5.74
19	wreath	11.23	documents	5.49
20	manger	10.99	controller	5.4
21	lute	10.78	cuyler	5.26
22	*vemeer*	10.61	fortninte	5.22
23	oversized	10.54	tricorn	4.85
24	accurate	10.47	dutton	4.68
25	*carvaggio*	10.28	potato	4.63
26	biblically	10.22	electronic	4.3
27	archimedes	10.08	decorations	4.16
28	lightning	9.53	masqua	3.71
29	35mm	9.5	calculus	3.68
30	lerapi	9.35	puck	3.38
31	laughing	9.2	switch	3.11
32	quick	9.2	greaves	3.04
33	drunkard	9.16	orrery	3.01
34	highcontrast	9.14	fisk	2.96
35	goatee	9.1	panelling	2.94
36	hephaestus	8.61	maths	2.84
37	chiaoscuro	8.49	shoot	2.7
38	wise	8.38	macaron	2.51
39	easter	8.36	contrat	2.49
40	*carravaggio*	8.04	whitened	2.43
41	marsterpiece	7.7	*2k*	2.33
42	iq	7.52	phtoto	2.29
43	saturn	7.51	roaming	2.25
44	rosace	7.39	boney	2.22
45	beret	7.17	haggis	2.15
46	beer	7.17	conservative	2.09
47	surgery	7.03	doran	2.07
48	sandals	7	lovecratian	2.06
49	skating	6.95	gnoll	1.96
50	corrected	6.82	pillhead	1.93
51	glory	6.32	antoinette	1.92
52	*1600s*	6.18	wellick	1.91
53	bw	6.07	tyrell	1.91
54	plague	6.04	floundering	1.9

55	laurels	6.04	renta	1.89
56	*wissing*	5.99	hgh	1.85
57	pluto	5.93	freemasons	1.82
58	*barocco*	5.88	*neoclassicist*	1.82
59	genesis	5.74	freyag	1.75
60	testament	5.63	boullee	1.63
61	*1614572159*	5.6	trophies	1.62
62	elche	5.51	medal	1.55
63	meaty	5.5	fever	1.54
64	jupiter	5.48	tricorne	1.53
65	alexandria	5.4	quiver	1.47
66	hades	5.35	*3000*	1.47
67	eaves	5.32	renault	1.45
68	macular	5.14	wakes	1.41
69	goofy	5.02	beber	1.38
70	ps	4.89	flex	1.37
71	iglesias	4.86	forsterling	1.35
72	trustworthy	4.85	kilt	1.34
73	*1615*	4.72	*portraiit*	1.33
74	scuta	4.71	*rokoko*	1.32
75	riccardi	4.66	bobbed	1.32
76	beaten	4.57	frampton	1.3
77	offering	4.53	meredit	1.3
78	apollo	4.41	showcase	1.29
79	xiv	4.33	keery	1.27
80	flemish	4.31	neuroscience	1.26
81	visors	4.24	inglaze	1.24
82	turrel	4.23	violencia	1.23
83	loots	4.08	waxed	1.22
84	wining	4.02	**letterboxing**	1.19
85	sus	4	ostrich	1.04
86	ruffle	3.95	cgi	1.03
87	ax	3.94	monarchy	1.02
88	**ringles**	3.94	cooked	1.01
89	degree	3.93	marquis	1.01
90	*16384k*	3.93	wreathed	1
91	drunk	3.86	gunslingers	0.99
92	honor	3.78	*40s*	0.98
93	furious	3.74	tholly	0.98
94	yawning	3.74	ade	0.97
95	nativity	3.71	santora	0.97
96	tobacco	3.69	oryantalist	0.97
97	zarathustra	3.67	sparring	0.96
98	thus	3.67	placards	0.96
99	spoke	3.66	dour	0.94
100	conundrum	3.64	ashigaru	0.92

Year	1800s		1900s	
RANK	Words	TF-IDF	Words	TF-IDF
1	photo	547.81	painting	1654.92
2	trees	386.78	oil	852.95
3	wearing	362.26	woman	850.82
4	river	356.76	red	792.7
5	dress	321.46	background	716.65
6	beautiful	280.29	blue	709.87
7	shot	271.62	view	658.3
8	girl	259.65	detail	642.39
9	walking	256.04	image	601.05
10	distance	251.47	style	568.77
11	old	248.09	sitting	540.06
12	boat	235.22	canvas	527.06
13	forest	191.54	drawing	525.52

14	profile	186.6	*abstract*	510.01
15	boy	183.59	yellow	502.84
16	foreground	179.74	detailed	497.23
17	shoulder	173.85	*portrait*	461.08
18	road	168.28	composition	453.27
19	pose	149.37	people	449.34
20	painted	139.7	grain	425.99
21	years	134.54	standing	404
22	sunny	127.67	picture	401.74
23	early	126.92	color	376.29
24	cropped	126.62	group	374.95
25	favorite	124.71	two	365.34
26	wikimedia	118.99	dark	352.68
27	symmetrical	118.11	looking	349.59
28	coat	116.06	table	348.56
29	cart	115.29	large	346.41
30	dressed	113.91	high	345.05
31	pic	109.44	scene	339.18
32	low	102.84	trending	333.42
33	couple	102.75	square	329.37
34	*640*	100.13	flowers	326.23
35	autumn	99.4	face	320.5
36	path	99.37	wikiart	319.28
37	farm	97.51	*landscape*	315.04
38	grass	96.58	full	312.12
39	wooded	95.66	head	309.94
40	photograph	94.67	front	303.03
41	sad	92.97	hat	295.23
42	phot	92.79	water	292.34
43	boats	92.63	side	281.65
44	commons	92.55	sky	281.18
45	rocky	90.42	city	279.64
46	seen	89.68	colors	277.93
47	sunset	88.99	stained	271.94
48	area	86.27	holding	265.64
49	nongraphic	86.11	chair	265.44
50	reddish	85.52	morning	265.07
51	dirt	85.41	tree	258.53
52	cute	84.92	1961	256.76
53	cutie	83.35	light	255.32
54	shaded	82.45	scheme	253.22
55	castle	82.06	hair	247.04
56	elegant	80.92	street	246.75
57	far	79.28	pink	244.89
58	jacket	78.86	colorful	244.04
59	peinture	78.1	orange	242.34
60	reading	77.67	unknown	240.8
61	carriage	77.39	ffffound	240.61
62	blonde	76.59	horse	240.5
63	*pitt*	71.68	body	237.98
64	horses	70.78	mountain	236.29
65	boys	70.54	vase	236.16
66	distant	70.43	room	234.48
67	official	70.35	purple	232.3
68	end	69.65	album	227.2
69	clothing	69.57	paper	224.01
70	online	68.74	artwork	218.72
71	famous	68.73	three	218.03
72	huile	67.94	mid	217.08
73	taken	67.38	dog	211.64
74	piggy	66.87	masterpiece	210.06

75	pixelated	66.15	building	206.18
76	valley	65.53	wide	205.57
77	stream	65.16	museum	202.92
78	grazing	65.06	house	202.85
79	**oilpainting**	63.51	courtesy	200.8
80	uniform	63.43	**artstastion**	199.39
81	masterful	62.33	*todorovitch*	196.38
82	picnic	62.1	women	195.01
83	look	61.55	ancient	190.17
84	expression	61.44	artists	188.7
85	exterior	61.41	deep	183.88
86	broadshouldered	61.28	illustration	181.08
87	bushes	60.27	made	181.02
88	fat	59.84	tumblr	180.91
89	random	59.07	cover	177.92
90	shore	59	details	177.57
91	epic	58.89	suit	175.01
92	late	58.26	russian	173.2
93	petite	58.12	panel	171.4
94	repoussoir	58.01	evening	167.12
95	cherry	57.24	lines	167.08
96	canopee	56.43	artlist	165.76
97	weather	56.23	soft	165.7
98	mittens	56.18	paint	165.4
99	clover	55.74	1921	164.9
100	devant	55.13	colours	163.49

Table S6. Summary statistics of regression performance for painting year prediction across 100 train-test splits. We trained 100 XGBoost regression models with different random seeds for each vector type. C-vectors show markedly superior performance across all metrics with minimal variation: mean $R^2 = 0.869 \pm 0.003$ and Pearson $\rho = 0.932 \pm 0.002$, compared to A-vectors' mean $R^2 = 0.202 \pm 0.004$ and Pearson $\rho = 0.450 \pm 0.005$.

A-vector

	**pearson**	**spearman**	**mae**	**rmse**	**r2**
mean	0.450489	0.478551	6.161701	9.364899	0.202408
std.dev.	0.005166	0.004986	0.020843	0.025757	0.004387
min	0.435114	0.464966	6.106355	9.284488	0.189268
25%	0.446549	0.475853	6.151085	9.349056	0.199159
50%	0.450601	0.478695	6.160301	9.365145	0.202372
75%	0.453714	0.481721	6.173639	9.383984	0.205110
max	0.466696	0.493860	6.215761	9.441761	0.216052

C-vector

	**pearson**	**spearman**	**mae**	**rmse**	**r2**
mean	0.932357	0.890857	2.311467	3.800089	0.868654
std.dev.	0.001625	0.001665	0.015774	0.043846	0.003030
min	0.927762	0.887760	2.275869	3.706918	0.860149
25%	0.931333	0.889805	2.301105	3.777689	0.866800
50%	0.932367	0.890523	2.311857	3.800240	0.868661
75%	0.933259	0.891771	2.323645	3.827070	0.870215
max	0.935804	0.895723	2.346094	3.921454	0.875032

Table S7. Summary statistics of classification performance for author and style prediction across 100 train-test splits. We trained 100 XGBoost models with different random seeds for each vector type (A-vector and C-vector) to classify 10 authors and 10 styles in Fig. 1. C-vectors demonstrate consistently superior performance across all metrics with low standard deviations, confirming robust classification capabilities compared to A-vectors.

Author

A-vector

	accuracy	accuracy (balanced)	f1 (macro)	f1 (weighted)	precision (macro)	recall (macro)	log loss
mean	0.583565599	0.326831355	0.331046405	0.546456068	0.436721611	0.326831355	1.32449586
std.dev.	0.008898177	0.006854847	0.008282018	0.009119711	0.033571256	0.006854847	0.030119962
min	0.554927809	0.30851122	0.312028464	0.517121104	0.35652634	0.30851122	1.268680153
25%	0.578782172	0.322274224	0.324444241	0.540578042	0.414560365	0.322274224	1.302388433
50%	0.583176397	0.325917217	0.329909863	0.546273006	0.437671639	0.325917217	1.322273316
75%	0.588826114	0.33063347	0.336112684	0.552382397	0.461101184	0.33063347	1.343891727
max	0.604519774	0.346487476	0.354700173	0.569653504	0.544645553	0.346487476	1.407261478

C-vector

	accuracy	accuracy (balanced)	f1 (macro)	f1 (weighted)	precision (macro)	recall (macro)	log loss
mean	0.953822976	0.822610201	0.855220523	0.952161272	0.918868774	0.822610201	0.150566892
std.dev.	0.004715654	0.022865878	0.027087948	0.004880699	0.049457981	0.022865878	0.012734639
min	0.945386064	0.769273334	0.802181936	0.943204333	0.846949079	0.769273334	0.12586882
25%	0.950408035	0.808596548	0.831478484	0.94885486	0.860770598	0.808596548	0.142485638
50%	0.953546767	0.821474167	0.861033968	0.951805846	0.952097573	0.821474167	0.150998086
75%	0.956214689	0.83761305	0.875265813	0.954866343	0.959948621	0.83761305	0.159781185
max	0.966101695	0.883834888	0.916910075	0.965549781	0.970353988	0.883834888	0.184305112

Style

A-vector

	accuracy	accuracy (balanced)	f1 (macro)	f1 (weighted)	precision (macro)	recall (macro)	log loss
mean	0.358330905	0.250726573	0.248907475	0.306640373	0.417627534	0.250726573	1.845044737
std.dev.	0.003132174	0.003200424	0.004200651	0.003431157	0.01198319	0.003200424	0.007103185
min	0.347734227	0.240945966	0.236669906	0.295522793	0.391834064	0.240945966	1.827056226
25%	0.356440332	0.248647343	0.246138381	0.304609063	0.409848307	0.248647343	1.840828052
50%	0.358334548	0.250485146	0.248789607	0.306659387	0.417156997	0.250485146	1.845287444
75%	0.360775171	0.25252282	0.251083068	0.308461929	0.425638368	0.25252282	1.849309708
max	0.367259216	0.259244106	0.259134844	0.315775543	0.455094689	0.259244106	1.868041779

C-vector

	accuracy	accuracy (balanced)	f1 (macro)	f1 (weighted)	precision (macro)	recall (macro)	log loss
mean	0.7625601	0.74950593	0.76456285	0.76058486	0.78649443	0.74950593	0.67424074
std.dev.	0.00315671	0.00378679	0.00360396	0.00325788	0.00366771	0.00378679	0.00855471
min	0.75666618	0.74192595	0.75729443	0.75449022	0.77810274	0.74192595	0.65730781
25%	0.76045461	0.74679554	0.76161153	0.75826924	0.78403324	0.74679554	0.67017865
50%	0.76264024	0.74974866	0.76481304	0.76058524	0.78652148	0.74974866	0.6732364
75%	0.7643159	0.75136828	0.76648481	0.76253652	0.78921797	0.75136828	0.67968573
max	0.77101851	0.76128081	0.77411015	0.76993925	0.79385258	0.76128081	0.69832445

References

1. Pharmapsychotic, Clip interrogator: Image to prompt with blip and clip (version 0.6.0) (https://github.com/pharmapsychotic/clip-interrogator) (2023).
2. HAAK Hammoud, et al., Synthclip: Are we ready for a fully synthetic clip training? *arXiv preprint arXiv:2402.01832* (2024).
